# Supplementary material for: The σB alternative sigma factor circuit modulates noise to generate different types of pulsing dynamics
Source: PLoS Comput Biol. 2023 Aug 4;19(8):e1011265. doi: 10.1371/journal.pcbi.1011265 (PMC10431680; doi:10.1371/journal.pcbi.1011265)
Supplement: S1 Fig — For each combination of stress magnitude (pstress) and noise amplitude (η) four simulations are shown (stress added at red dashed line, t = 0). Parameter values and other details on simulation conditions for this figure are described in S1 Table. (PDF) [file pcbi.1011265.s001.pdf]

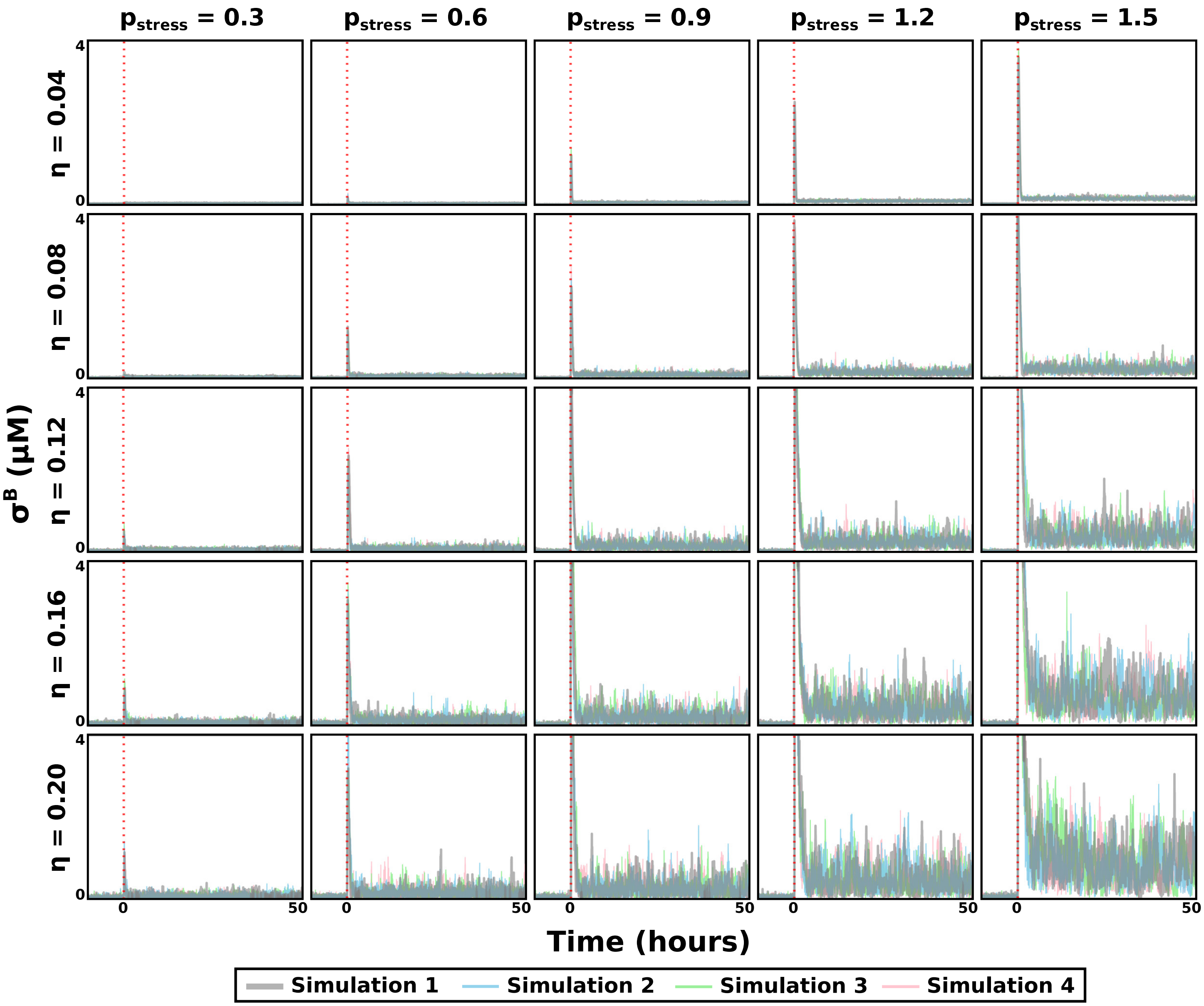

S Fig 1. Stochastic pulsing is not achievable in the model by tuning η alone. For each combination of stress magnitude (p_{stress}) and noise amplitude (η) four simulations are shown (stress added at red dashed line, $t = 0$). Parameter values and other details on simulation conditions for this figure are described in S1 Table.
