# Supplementary material for: The σB alternative sigma factor circuit modulates noise to generate different types of pulsing dynamics
Source: PLoS Comput Biol. 2023 Aug 4;19(8):e1011265. doi: 10.1371/journal.pcbi.1011265 (PMC10431680; doi:10.1371/journal.pcbi.1011265)
Supplement: S2 Fig — (A) Bifurcation diagrams for the various parameters, each plot shows three diagrams (for the varying stress levels pstress = 0.05 μM, 0.20 μM, and 0.80 μM). The stars mark the parameter value for the original Narula model. Each x-axis is log10 scaled, and if the parameter’s original value is p0, it is varied over the range (p0/10, 10p0), corresponding to a tenfold decrease and increase in the target parameter, respectively. Only by tuning kK2 or λW can instability be achieved. For some parameters (kK2, kP, and F), the curve for pstress = 0.80 μM reaches much larger values compared to the other two curves, making these hard to distinguish. To avoid figure crowding, periodic orbits are not displayed in these diagrams, however, they are instead shown in S3 Fig. (B) Bifurcation diagram for the parameter pstress (the magnitude of the stress) over the interval (0.1 μM,10.0 μM), with the x-axis log10 scaled. Instability cannot be produced by tuning pstress only. Parameter values and other details on simulation conditions for this figure are described in S1 Table. (PDF) [file pcbi.1011265.s002.pdf]

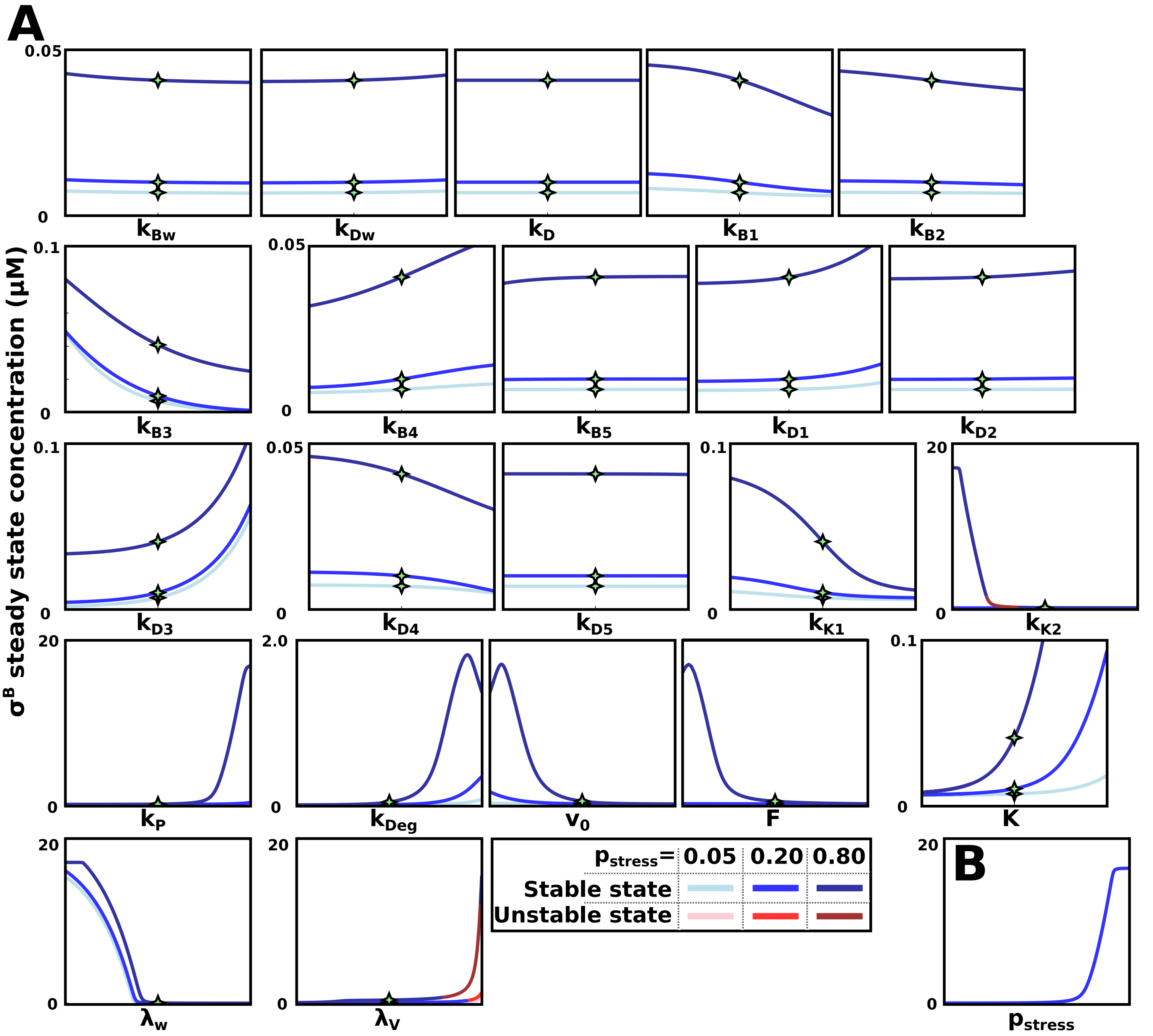

S Fig 2. Only through the tuning of two parameters can oscillations be achieved. (A) Bifurcation diagrams for the various parameters, each plot shows three diagrams (for the varying stress levels $p_{stress} = 0.05$ µM, 0.20 µM, and 0.80 µM). The stars mark the parameter value for the original Narula model. Each x-axis is log10 scaled, and if the parameter's original value is p_0, it is varied over the range $(p_0/10, 10p_0)$, corresponding to a tenfold decrease and increase in the target parameter, respectively. Only by tuning k_{K2} or λ_W can instability be achieved. For some parameters (k_{K2}, k_P, and F), the curve for $p_{stress} = 0.80$ µM reaches much larger values compared to the other two curves, making these hard to distinguish. To avoid figure crowding, periodic orbits are not displayed in these diagrams, however, they are instead shown in S3 Fig. (B) Bifurcation diagram for the parameter p_{stress} (the magnitude of the stress) over the interval (0.1 µM,10.0 µM), with the x-axis log10 scaled. Instability cannot be produced by tuning p_{stress} only. Parameter values and other details on simulation conditions for this figure are described in S1 Table.
