# Supplementary material for: The σB alternative sigma factor circuit modulates noise to generate different types of pulsing dynamics
Source: PLoS Comput Biol. 2023 Aug 4;19(8):e1011265. doi: 10.1371/journal.pcbi.1011265 (PMC10431680; doi:10.1371/journal.pcbi.1011265)
Supplement: S3 Fig — Four of the bifurcation diagrams in S2 Fig display periodic orbits, these are shown in more details here. The stars mark the parameters’ values for the original Narula model and all x-axes are log10 scaled. (A) Bifurcation diagram with respect to the parameter kK2. (B-D) Bifurcation diagram with respect to the parameter λV, for three different values of pstress. Note that for these the y-axes are log10 scaled (to help show the periodic orbits more clearly), unlikely in S2 Fig where they are linearly scaled. Parameter values and other details on simulation conditions for this figure are described in S1 Table. (PDF) [file pcbi.1011265.s003.pdf]

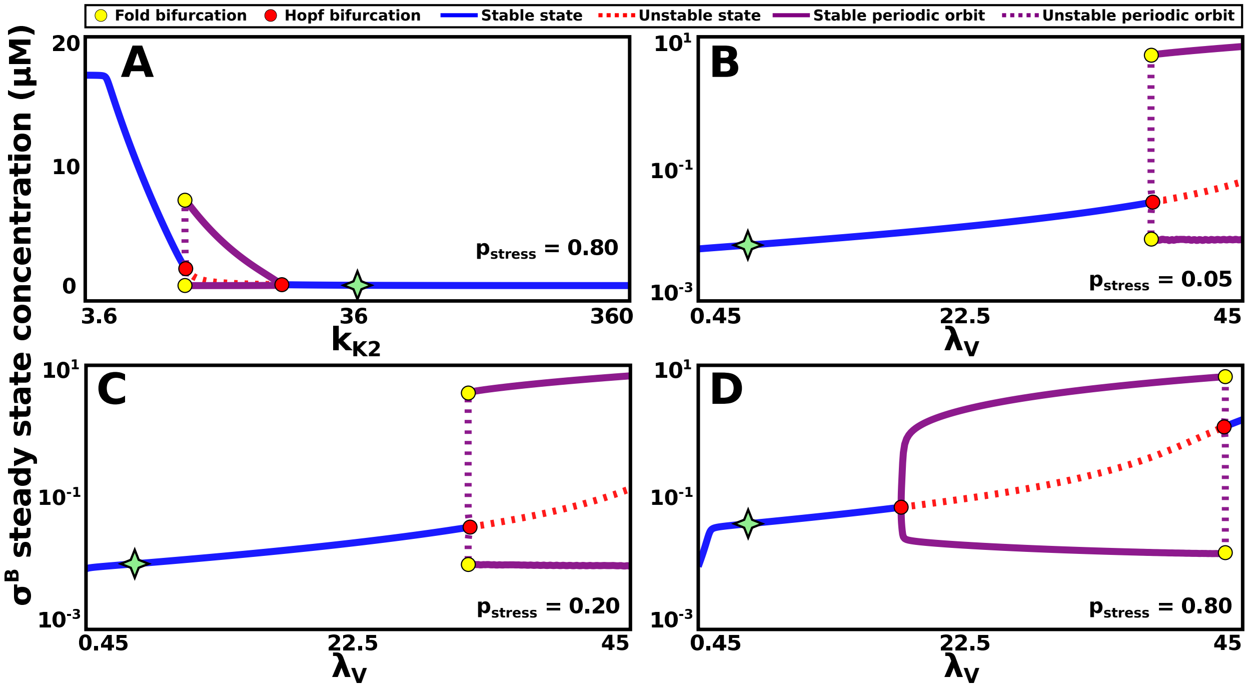

S Fig 3. Bifurcation diagrams with periodic orbits shown. Four of the bifurcation diagrams in S2 Fig display periodic orbits, these are shown in more details here. The stars mark the parameters' values for the original Narula model and all x-axes are log10 scaled. (A) Bifurcation diagram with respect to the parameter k_{K2}. (B-D) Bifurcation diagram with respect to the parameter λ_V, for three different values of p_{stress}. Note that for these the y-axes are log10 scaled (to help show the periodic orbits more clearly), unlikely in S2 Fig where they are linearly scaled. Parameter values and other details on simulation conditions for this figure are described in S1 Table.
