# Supplementary material for: The σB alternative sigma factor circuit modulates noise to generate different types of pulsing dynamics
Source: PLoS Comput Biol. 2023 Aug 4;19(8):e1011265. doi: 10.1371/journal.pcbi.1011265 (PMC10431680; doi:10.1371/journal.pcbi.1011265)
Supplement: S4 Fig — Here we test whether removing all noise from phosphatase reactions can still allow both response dynamics. In Fig 2 we demonstrate that both system behaviours can be recreated even as phosphatase levels remain constant (unlike in [45] which assumed noisy concentrations of P). To do this, we use the CLE. The CLE adds noise to reaction channels, rather than species concentrations (with the former affecting the latter). This means that there’s still noise in P due to noise in the reactions involving P. (A,B) Here we remove noise from all reactions involving P (and components containing P). To do this, we use our modified Narula model that allows us to tune upstream noise (Section 4.2) and set ηamp = 0.0. Next, we recreate both the single response pulse (A, pprod = 50.0, η = 0.01) and stochastic pulsing (B, pprod = 25.0, η = 0.09) behaviours. This demonstrates that both behaviours can be generated by the core circuit, and are not dependent on any form of upstream noise. Stress is added at red dashed lines (t = 0) and each plot shows four simulations. Full parameter sets for this figure are described in S6 Table. (PDF) [file pcbi.1011265.s004.pdf]

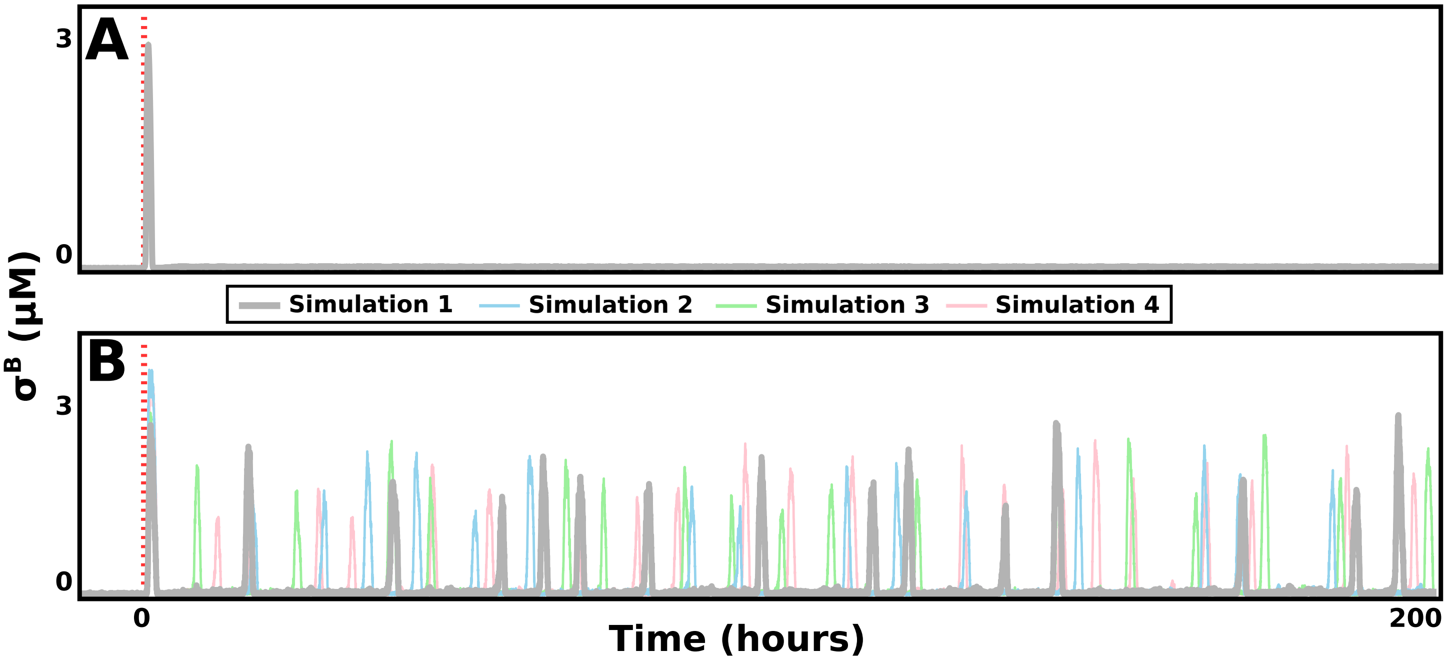

S Fig 4. Both behaviours can be reproduced even as we remove all noise from upstream reactions. Here we test whether removing all noise from phosphatase reactions can still allow both response dynamics. In Fig 2 we demonstrate that both system behaviours can be recreated even as phosphatase levels remain constant (unlike in Narula (2016) which assumed noisy concentrations of P). To do this, we use the CLE. The CLE adds noise to reaction channels, rather than species concentrations (with the former affecting the latter). This means that there's still noise in P due to noise in the reactions involving P. (A,B) Here we remove noise from all reactions involving P (and components containing P). To do this, we use our modified Narula model that allows us to tune upstream noise (Section 4.2) and set $\eta_{amp} = 0.0$. Next, we recreate both the single response pulse (A, $p_{prod} = 50.0$, $\eta = 0.01$) and stochastic pulsing (B, $p_{prod} = 25.0$, $\eta = 0.09$) behaviours. This demonstrates that both behaviours can be generated by the core circuit, and are not dependent on any form of upstream noise. Stress is added at red dashed lines ($t = 0$) and each plot shows four simulations. Full parameter sets for this figure are described in S6 Table.
