# Supplementary material for: The σB alternative sigma factor circuit modulates noise to generate different types of pulsing dynamics
Source: PLoS Comput Biol. 2023 Aug 4;19(8):e1011265. doi: 10.1371/journal.pcbi.1011265 (PMC10431680; doi:10.1371/journal.pcbi.1011265)
Supplement: S5 Fig — (A) In the time leading up to a stochastic pulse, the total amount of σB (blue, scale marked at the left) and RsbW (blue, scale marked at the right) is plotted. Four simulations are shown. (B) For the same four simulations, the system is shown in σB-RsbW phase space. The state at the beginning of the simulation is marked with a blue dot. Just before the pulse is initiated (σB levels start to rise), the total RsbW concentration dips. Although harder to see, the dip can also be distinguished in (A). Parameter values and other details on simulation conditions for this figure are described in S1 Table. (PDF) [file pcbi.1011265.s005.pdf]

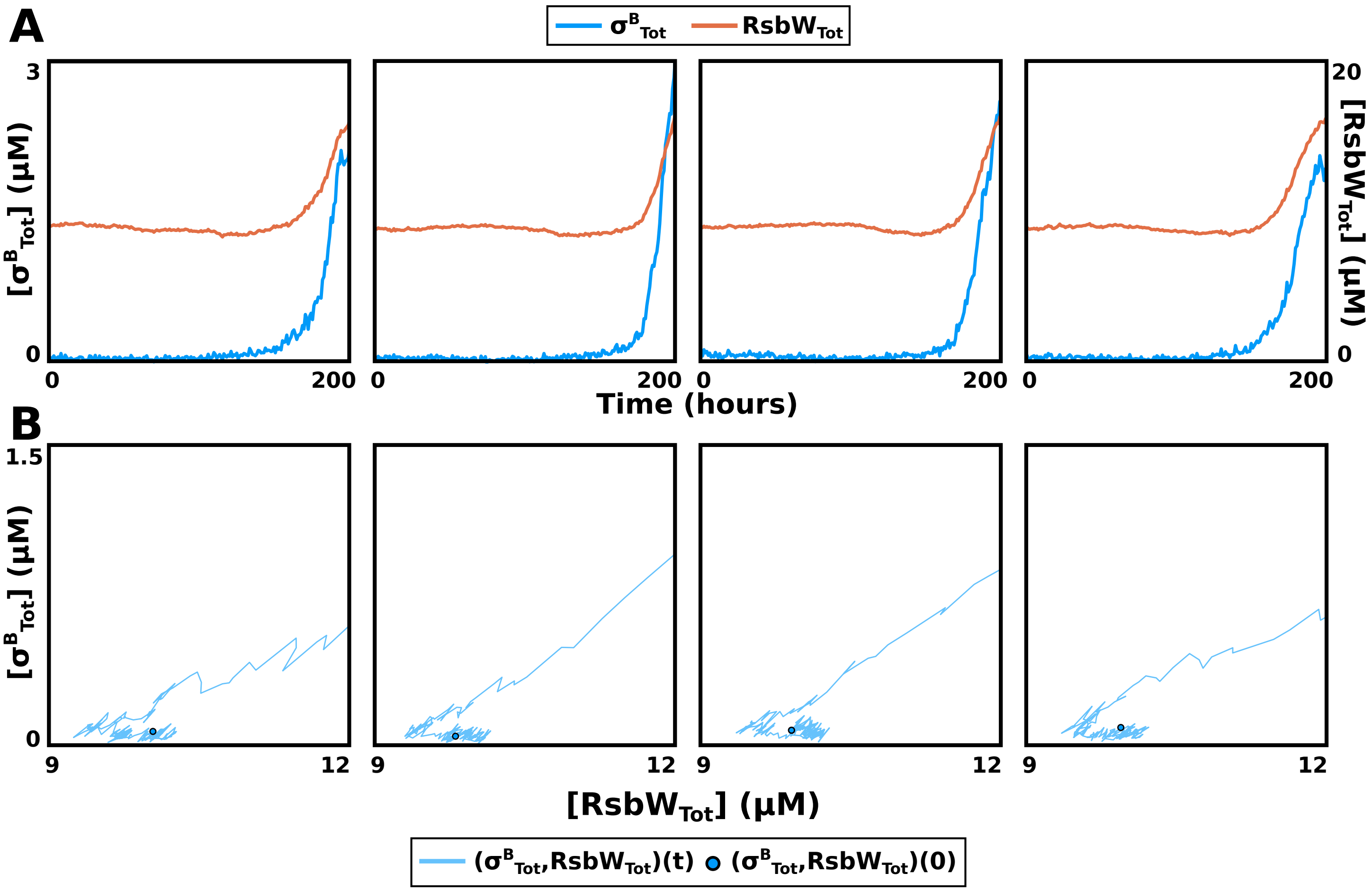

S Fig 5. The stochastic pulses in σ^B are preceded by a reduction in total RsbW concentration. (A) In the time leading up to a stochastic pulse, the total amount of σ^B (blue, scale marked at the left) and RsbW (blue, scale marked at the right) is plotted. Four simulations are shown. (B) For the same four simulations, the system is shown in σ^B-RsbW phase space. The state at the beginning of the simulation is marked with a blue dot. Just before the pulse is initiated (σ^B levels start to rise), the total RsbW concentration dips. Although harder to see, the dip can also be distinguished in (A). Parameter values and other details on simulation conditions for this figure are described in S1 Table.
