# Supplementary material for: The σB alternative sigma factor circuit modulates noise to generate different types of pulsing dynamics
Source: PLoS Comput Biol. 2023 Aug 4;19(8):e1011265. doi: 10.1371/journal.pcbi.1011265 (PMC10431680; doi:10.1371/journal.pcbi.1011265)
Supplement: S6 Fig — We find the two functions Msrp(p) and Msp(p) of our target parameter (this example used pstress) (Section 4.5.2). We define three areas: Asrp is the area which is beneath the Msrp(p) curve but above Msp(p), with Asp defined similarly. We also define Asrp,sp as the area which is beneath both curves. Finally, our measure is defined as Dsrp,sp(p)=Asrp·AspAsrp+Asrp,sp+Asp. Parameter values and other details on simulation conditions for this figure are described in S2 Table. (PDF) [file pcbi.1011265.s006.pdf]

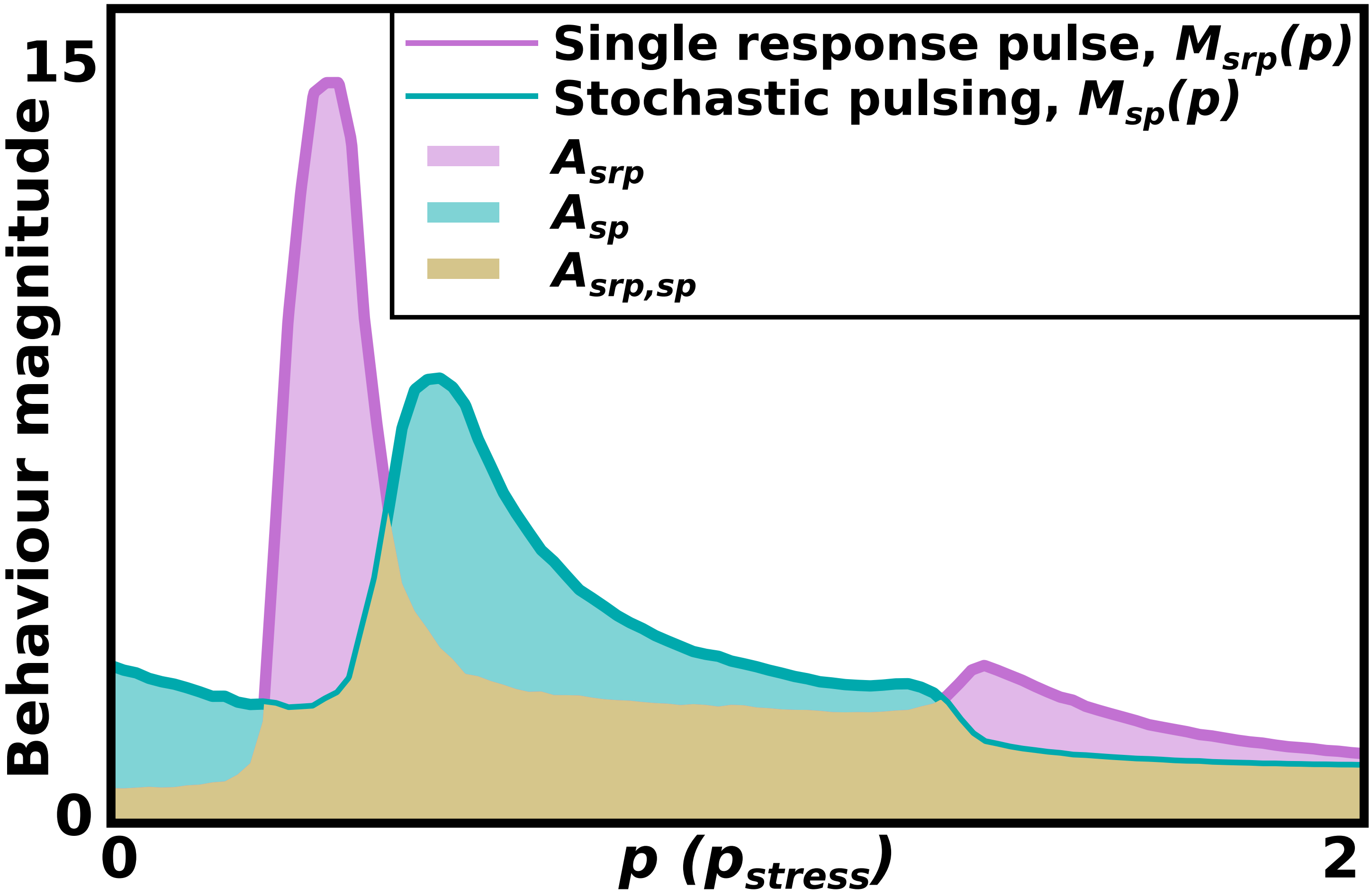

S Fig 6. A measure of the system's ability to generate both behaviours distinctly as a single parameter is tuned. We find the two functions $M_{srp}(p)$ and $M_{sp}(p)$ of our target parameter (this example used p_{stress}) (Section 4.5.2). We define three areas: A_{srp} is the area which is beneath the $M_{srp}(p)$ curve but above $M_{sp}(p)$, with A_{sp} defined similarly. We also define $A_{srp,sp}$ as the area which is beneath both curves. Finally, our measure is defined as $D_{srp,sp}(p) = \frac{\sqrt{A_{srp} \cdot A_{sp}}}{A_{srp}+A_{srp,sp}+A_{sp}}$. Parameter values and other details on simulation conditions for this figure are described in S2 Table.
