# Supplementary material for: The σB alternative sigma factor circuit modulates noise to generate different types of pulsing dynamics
Source: PLoS Comput Biol. 2023 Aug 4;19(8):e1011265. doi: 10.1371/journal.pcbi.1011265 (PMC10431680; doi:10.1371/journal.pcbi.1011265)
Supplement: S7 Fig — The function Dsrp,sp(pstress,p¯) measures the system’s ability to distinctly generate both behaviours, while varying the parameter pstress only (Section 4.5.2). The optimal value, (kK2, η) = (7.0hr−1, 0.025), is found close to the bottom left corner (light green dot). We chose the upper limit of η (0.15) as beyond this point behaviours start to become obscured by noise levels. Parameter values and other details on simulation conditions for this figure are described in S2 Table. (PDF) [file pcbi.1011265.s007.pdf]

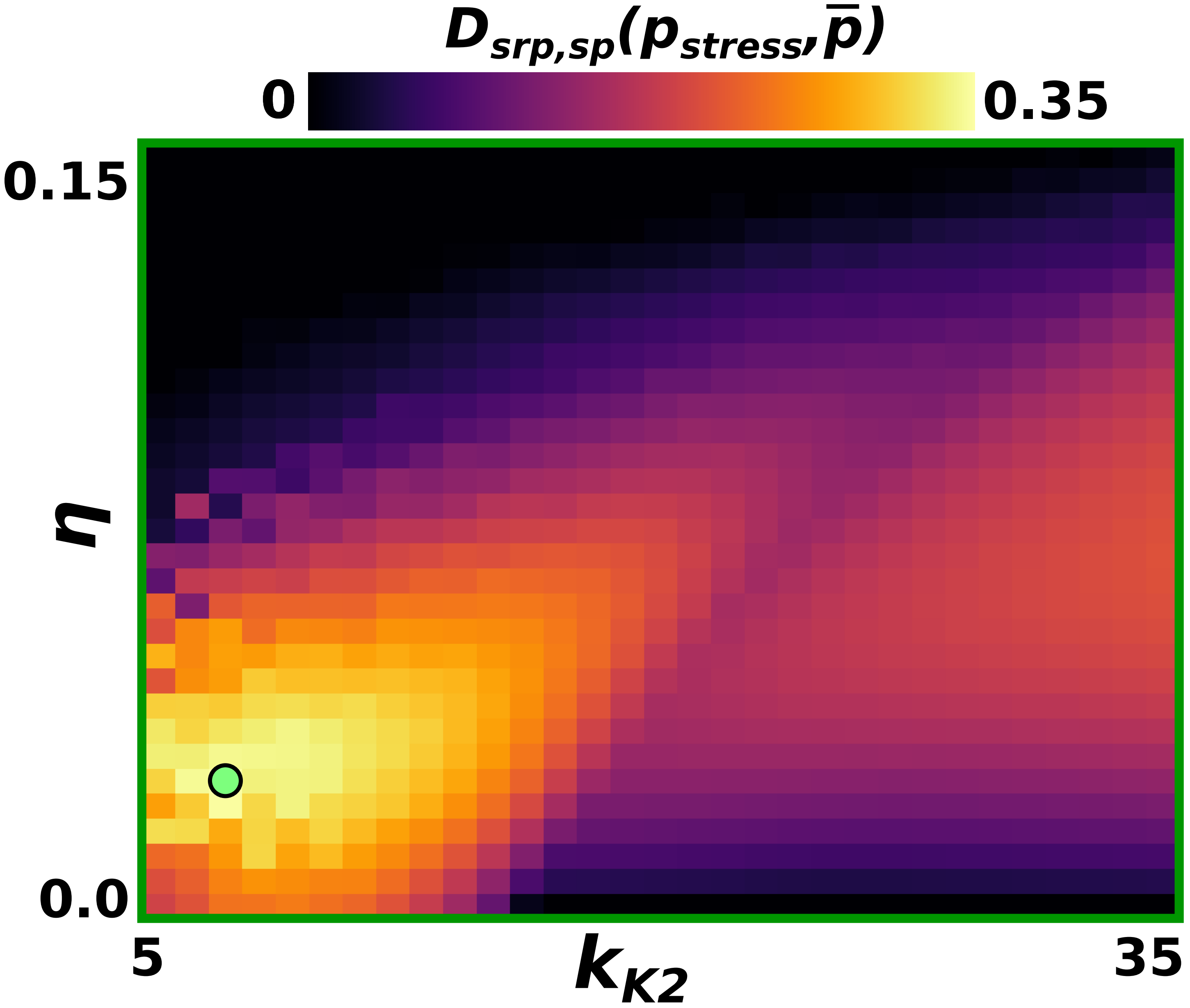

S Fig 7. Heatmap showing which combinations of k_{K2} and η enables the system to optimally generate both behaviours. The function $D_{srp,sp}(p_{stress}, \bar{p})$ measures the system's ability to distinctly generate both behaviours, while varying the parameter p_{stress} only (Section 4.5.2). The optimal value, $(k_{K2}, \eta) = (7.0hr^{-1}, 0.025)$, is found close to the bottom left corner (light green dot). We chose the upper limit of η (0.15) as beyond this point behaviours start to become obscured by noise levels. Parameter values and other details on simulation conditions for this figure are described in S2 Table.
