# Supplementary material for: The σB alternative sigma factor circuit modulates noise to generate different types of pulsing dynamics
Source: PLoS Comput Biol. 2023 Aug 4;19(8):e1011265. doi: 10.1371/journal.pcbi.1011265 (PMC10431680; doi:10.1371/journal.pcbi.1011265)
Supplement: S8 Fig — (A,B) The system’s response to stress (red dashed line, t = 0), for (kK2, η) = (7.0hr−1, 0.025) and pstress = 0.24 μM (A) and pstress = 0.28 μM (B). Each plot shows four simulations. Parameter values and other details on simulation conditions for this figure are described in S1 Table. (PDF) [file pcbi.1011265.s008.pdf]

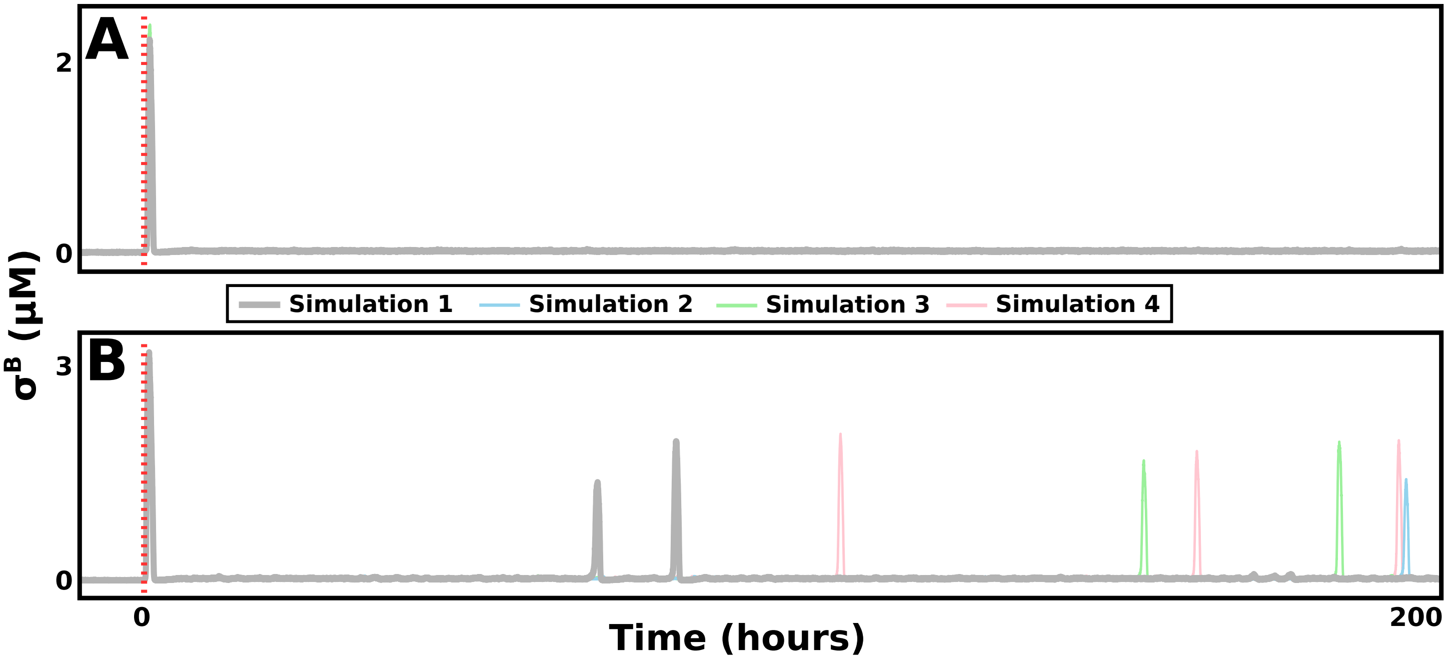

S Fig 8. An optimised parameter set is able to generate both behaviours by varying p_{stress} only. (A,B) The system's response to stress (red dashed line, $t = 0$), for $(k_{K2}, \eta) = (7.0hr^{-1}, 0.025)$ and $p_{stress} = 0.24$ µM (A) and $p_{stress} = 0.28$ µM (B). Each plot shows four simulations. Parameter values and other details on simulation conditions for this figure are described in S1 Table.
