# Supplementary material for: The σB alternative sigma factor circuit modulates noise to generate different types of pulsing dynamics
Source: PLoS Comput Biol. 2023 Aug 4;19(8):e1011265. doi: 10.1371/journal.pcbi.1011265 (PMC10431680; doi:10.1371/journal.pcbi.1011265)
Supplement: S9 Fig — Heatmaps showing the two behaviours occurrences in kB5-kD5-space. In all plots, the behaviours region of occurrence is similar to a kP ⋅ pstress = C curve. Parameter values and other details on simulation conditions for this figure are described in S2 Table. (PDF) [file pcbi.1011265.s009.pdf]

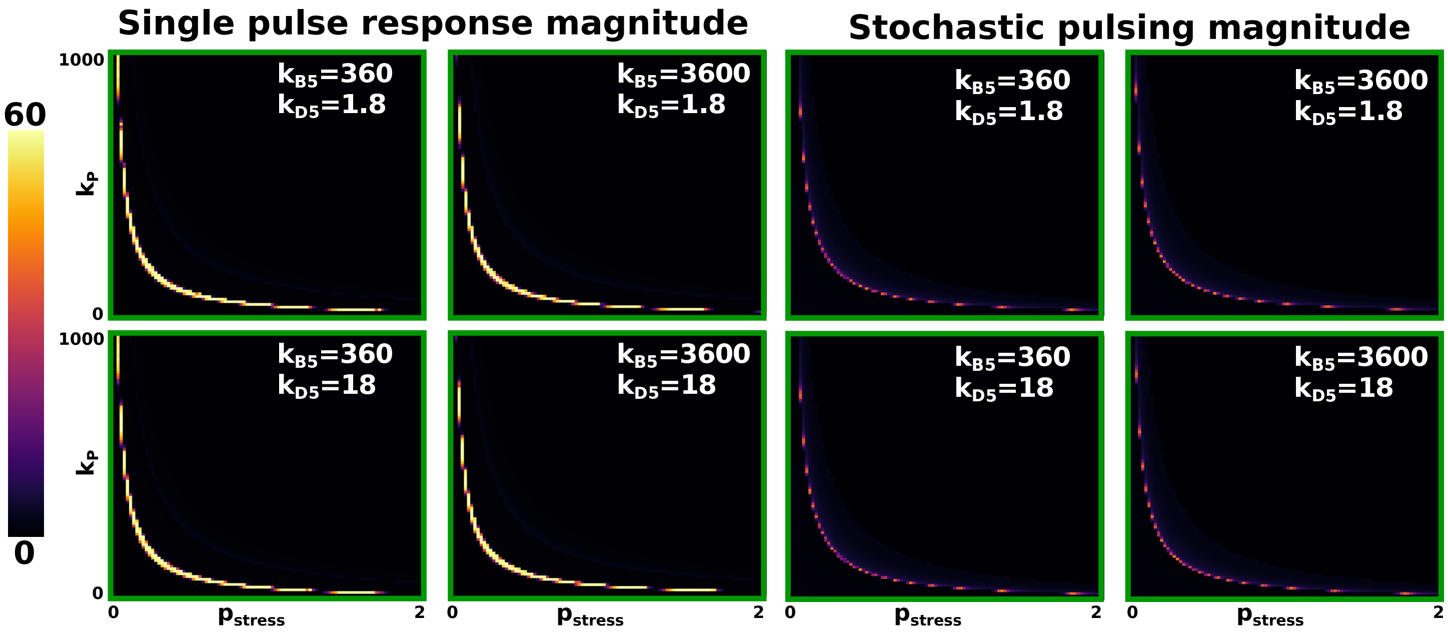

S Fig 9. The shape of the two behaviours' regions of occurrence is stable as k_{B5} and k_{D5} are changed. Heatmaps showing the two behaviours occurrences in k_{B5}-k_{D5}-space. In all plots, the behaviours region of occurrence is similar to a $k_P \cdot p_{stress} = C$ curve. Parameter values and other details on simulation conditions for this figure are described in S2 Table.
