# Supplementary material for: The σB alternative sigma factor circuit modulates noise to generate different types of pulsing dynamics
Source: PLoS Comput Biol. 2023 Aug 4;19(8):e1011265. doi: 10.1371/journal.pcbi.1011265 (PMC10431680; doi:10.1371/journal.pcbi.1011265)
Supplement: S10 Fig — Each heatmap describes the behaviour’s magnitude as the parameters pprod (x-axis) and pfrac (y-axis) are varied. A total of 36 heatmaps are plotted and placed in a 6x6 grid for a range of values of kB5 and kD5. There is a distinct spike in magnitude as pprod is varied. Changes to pfrac, pprod, and kD5 all have some effect on the magnitude, but not as distinct as changes to pprod. Parameter values and other details on simulation conditions for this figure are described in S5 Table. (PDF) [file pcbi.1011265.s010.pdf]

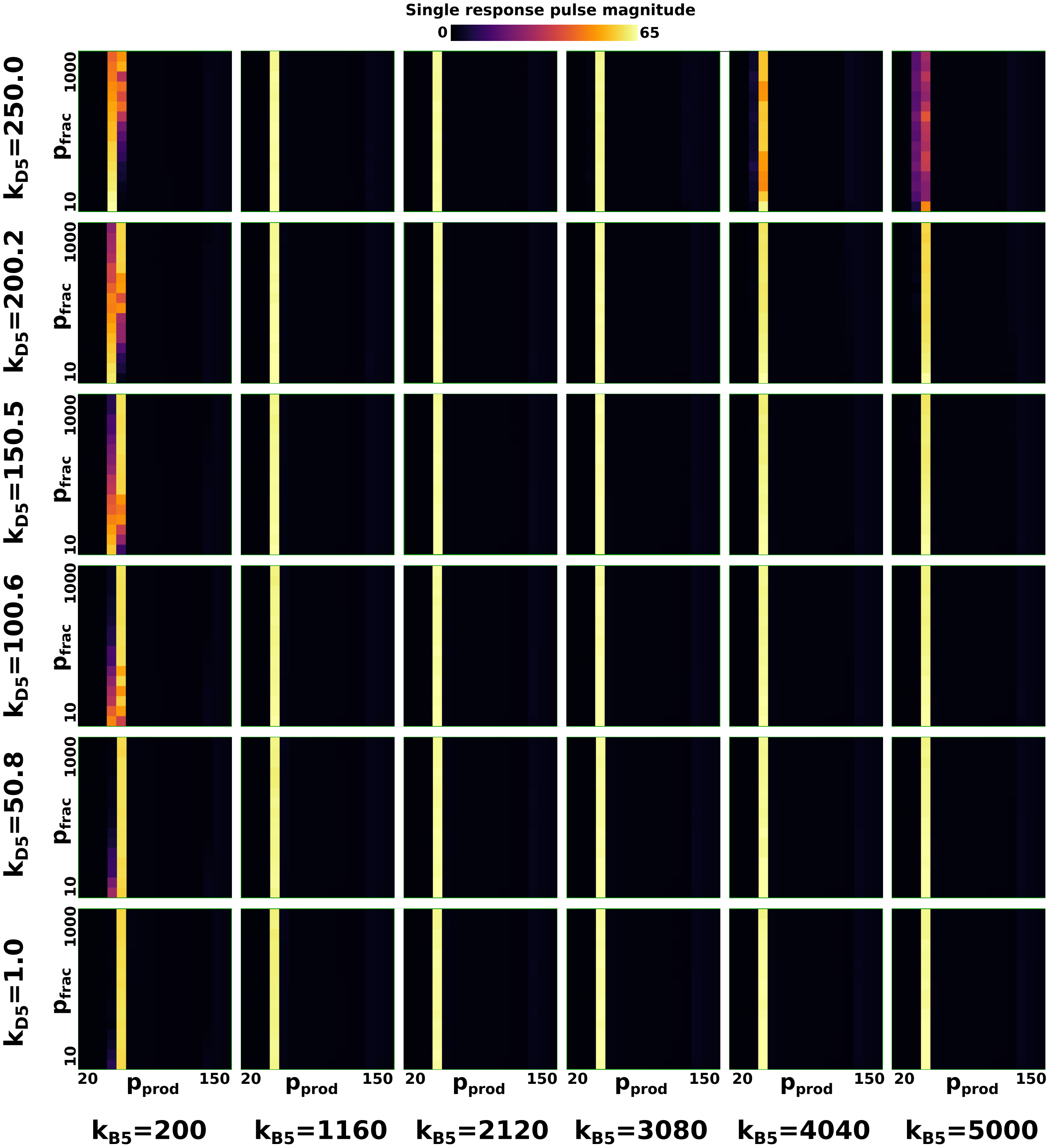

S Fig 10. Heatmaps describing the magnitude of the single response pulse behaviour for various values of k_{B5} and k_{D5}. Each heatmap describes the behaviour's magnitude as the parameters p_{prod} (x-axis) and p_{frac} (y-axis) are varied. A total of 36 heatmaps are plotted and placed in a 6x6 grid for a range of values of k_{B5} and k_{D5}. There is a distinct spike in magnitude as p_{prod} is varied. Changes to p_{frac}, p_{prod}, and k_{D5} all have some effect on the magnitude, but not as distinct as changes to p_{prod}. Parameter values and other details on simulation conditions for this figure are described in S5 Table.
