# Supplementary material for: The σB alternative sigma factor circuit modulates noise to generate different types of pulsing dynamics
Source: PLoS Comput Biol. 2023 Aug 4;19(8):e1011265. doi: 10.1371/journal.pcbi.1011265 (PMC10431680; doi:10.1371/journal.pcbi.1011265)
Supplement: S13 Fig — Each heatmap describes the behaviour’s magnitude as the parameters kD5 (x-axis) and kB5 (y-axis) are varied. A total of 36 heatmaps are plotted and placed in a 6x6 grid for a range of values of pprod and pfrac. Only for one value of pprod do changes in the other parameters have a major effect on the behaviour’s magnitude. Parameter values and other details on simulation conditions for this figure are described in S5 Table. (PDF) [file pcbi.1011265.s013.pdf]

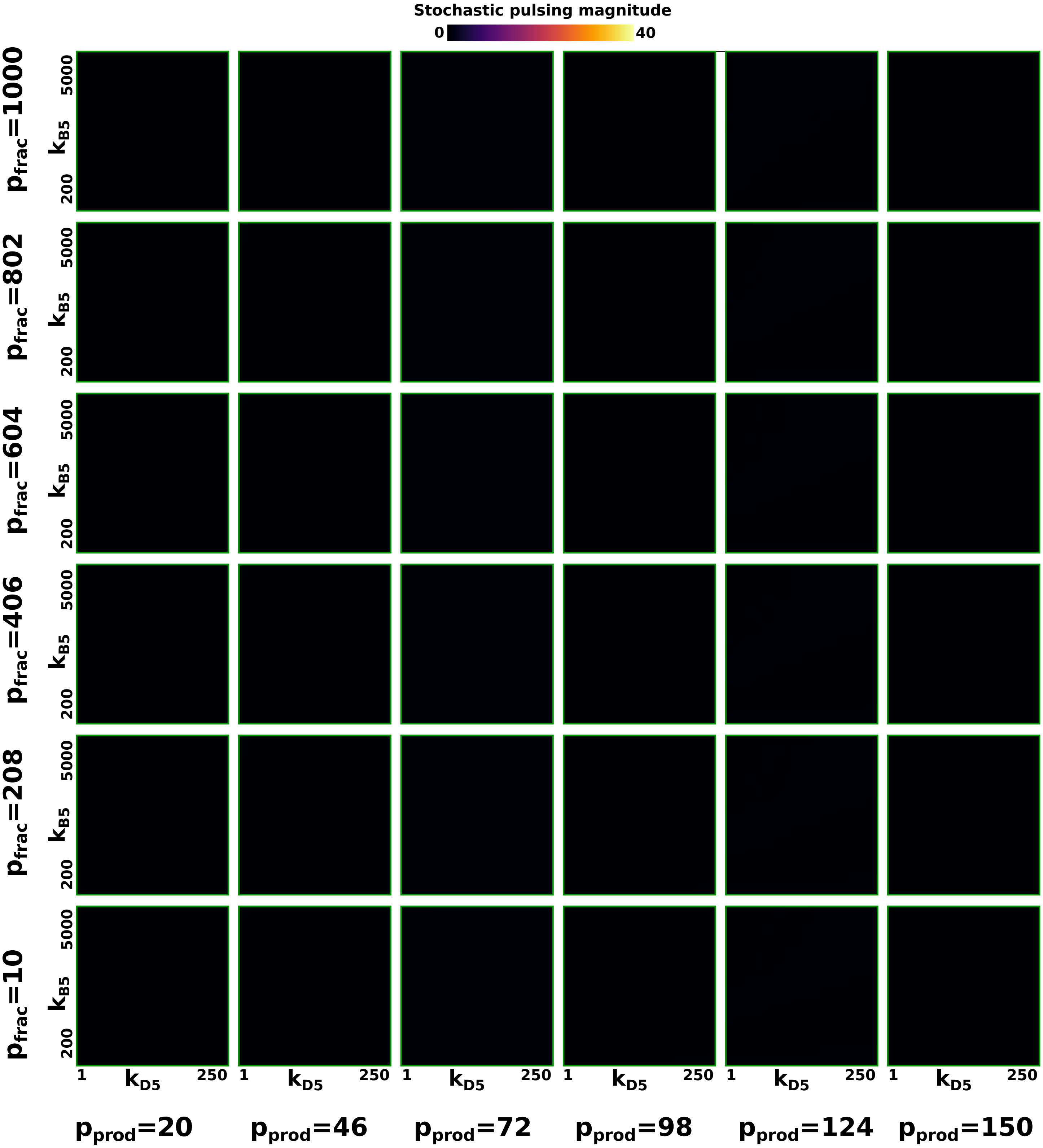

S Fig 13. Heatmaps describing the magnitude of the stochastic pulsing response behaviour for various values of p_{prod} and p_{frac}. Each heatmap describes the behaviour's magnitude as the parameters k_{D5} (x-axis) and k_{B5} (y-axis) are varied. A total of 36 heatmaps are plotted and placed in a 6x6 grid for a range of values of p_{prod} and p_{frac}. Only for one value of p_{prod} do changes in the other parameters have a major effect on the behaviour's magnitude. Parameter values and other details on simulation conditions for this figure are described in S5 Table.
