# Supplementary material for: The σB alternative sigma factor circuit modulates noise to generate different types of pulsing dynamics
Source: PLoS Comput Biol. 2023 Aug 4;19(8):e1011265. doi: 10.1371/journal.pcbi.1011265 (PMC10431680; doi:10.1371/journal.pcbi.1011265)
Supplement: S14 Fig — Each heatmap describes the behaviour’s magnitude as the parameters pprod (x-axis) and kB5 (y-axis) are varied. A total of 36 heatmaps are plotted and placed in a 6x6 grid for a range of values of pfrac and kD5. There is a distinct spike in magnitude as pprod is varied. Changes to pfrac, pprod, and kD5 have little effect on the behaviour’s magnitude. Parameter values and other details on simulation conditions for this figure are described in S5 Table. (PDF) [file pcbi.1011265.s014.pdf]

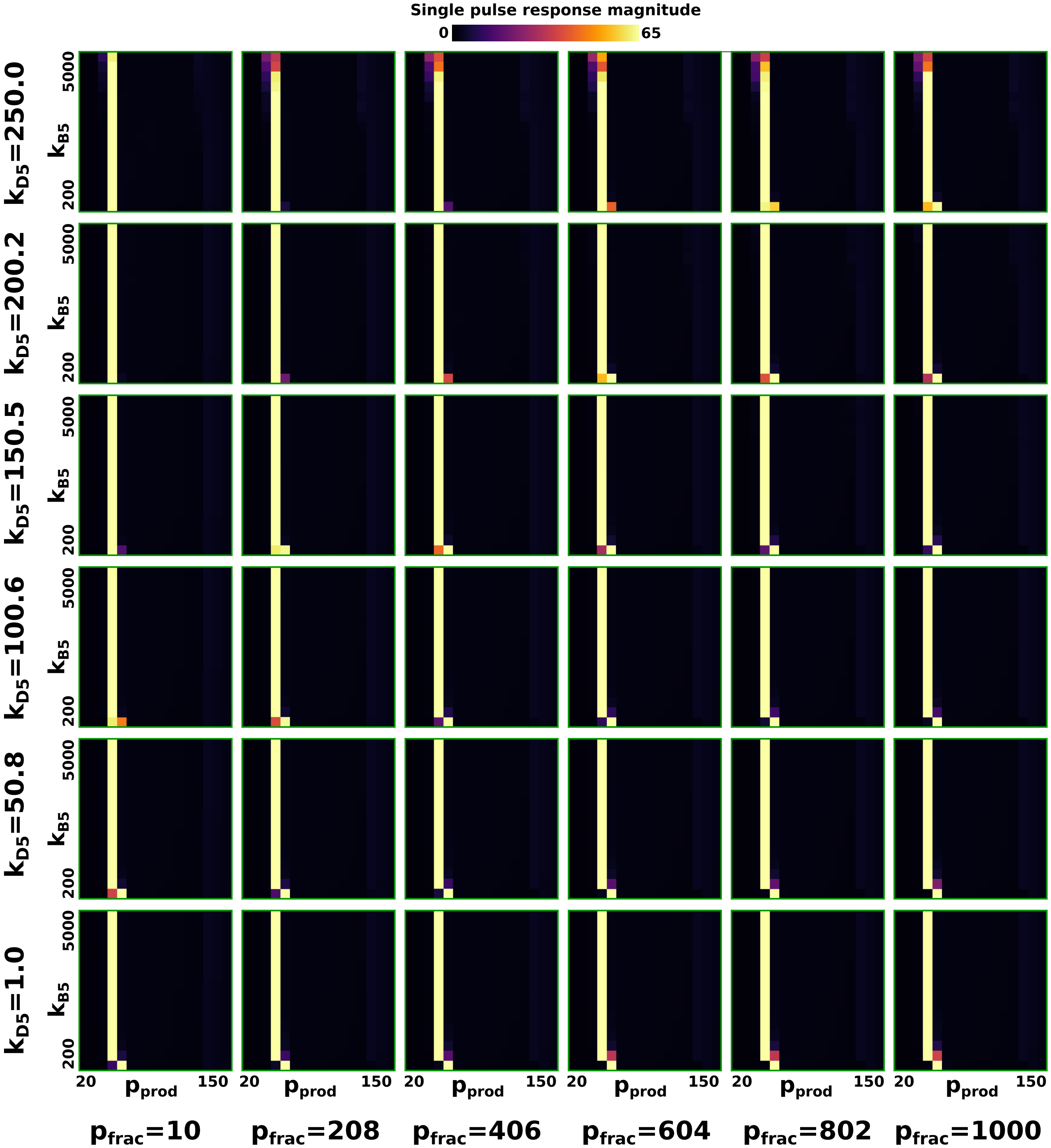

S Fig 14. Heatmaps describing the magnitude of the single response pulse behaviour for various values of p_{frac} and k_{D5}. Each heatmap describes the behaviour's magnitude as the parameters p_{prod} (x-axis) and k_{B5} (y-axis) are varied. A total of 36 heatmaps are plotted and placed in a 6x6 grid for a range of values of p_{frac} and k_{D5}. There is a distinct spike in magnitude as p_{prod} is varied. Changes to p_{frac}, p_{prod}, and k_{D5} have little effect on the behaviour's magnitude. Parameter values and other details on simulation conditions for this figure are described in S5 Table.
