# Supplementary material for: The σB alternative sigma factor circuit modulates noise to generate different types of pulsing dynamics
Source: PLoS Comput Biol. 2023 Aug 4;19(8):e1011265. doi: 10.1371/journal.pcbi.1011265 (PMC10431680; doi:10.1371/journal.pcbi.1011265)
Supplement: S16 Fig — Subplot A is identical to subplot A of Fig 4. Subplots C-N are identical to subplots B-M of Fig 4. Please see Fig 4 for legends of these subplots. (B) Bifurcation diagram, showing the system’s steady state as a function of pprod. After a region of inactivity, the steady state becomes unstable (implying a limit cycle). As pprod increases further, the steady state becomes stable, with an increasing concentration that eventually saturates. The transition in the bifurcation diagram corresponds to the transition in C-N (stability at an inactive state, a limit cycle, stability at an active state). We note that the bifurcation diagram is computed from the deterministic (ODE) system, while subfigures A, and C-N all use the stochastic (SDE) system. Adding noise to a non-linear system affects the parameter values at which stability occurs. This explains that the region of instability in (B) occurs for different pprod values as compared to A. While the deterministic bifurcation analysis in B cannot be directly translated to our stochastic system, it is still worth noting this similarity between the two cases. Parameter values and other details on simulation conditions for this figure are described in S5 Table. (PDF) [file pcbi.1011265.s016.pdf]

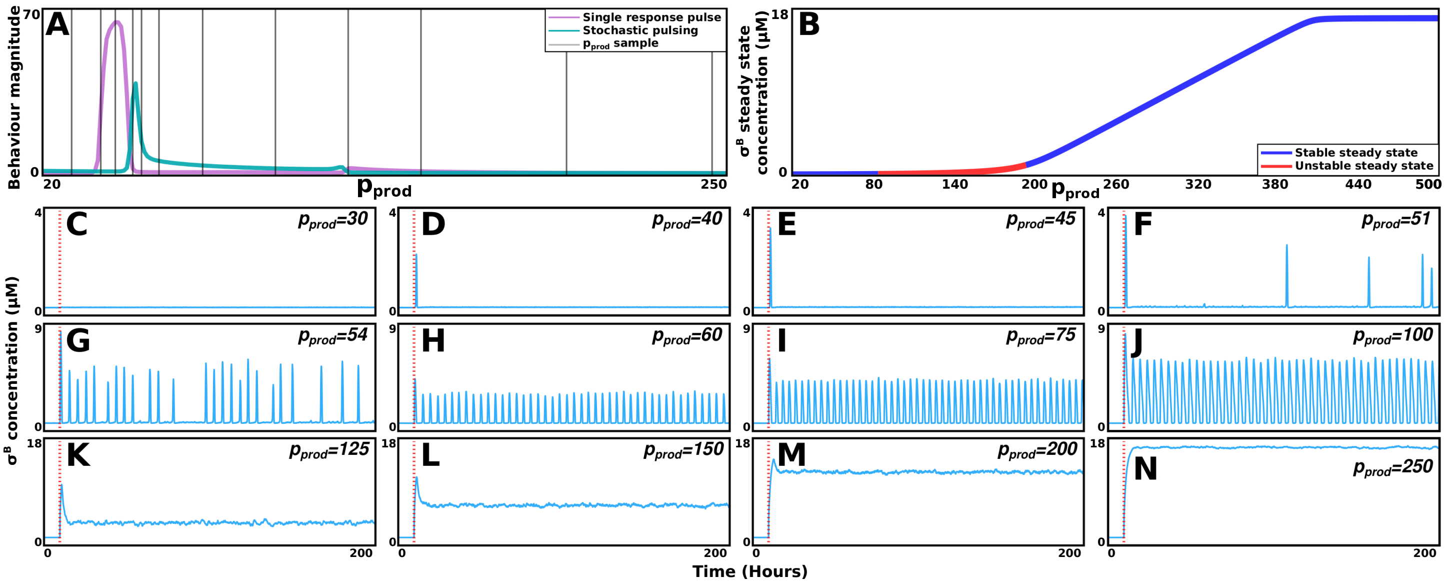

S Fig 16. Fig 4 with a system bifurcation diagram added. Subplot A is identical to subplot A of Fig 4. Subplots C-N are identical to subplots B-M of Fig 4. Please see Fig 4 for legends of these subplots. (B) Bifurcation diagram, showing the system's steady state as a function of p_{prod}. After a region of inactivity, the steady state becomes unstable (implying a limit cycle). As p_{prod} increases further, the steady state becomes stable, with an increasing concentration that eventually saturates. The transition in the bifurcation diagram corresponds to the transition in C-N (stability at an inactive state, a limit cycle, stability at an active state). We note that the bifurcation diagram is computed from the deterministic (ODE) system, while subfigures A, and C-N all use the stochastic (SDE) system. Adding noise to a non-linear system affects the parameter values at which stability occurs. This explains that the region of instability in (B) occurs for different p_{prod} values as compared to A. While the deterministic bifurcation analysis in B cannot be directly translated to our stochastic system, it is still worth noting this similarity between the two cases. Parameter values and other details on simulation conditions for this figure are described in S5 Table.
