# Supplementary material for: The σB alternative sigma factor circuit modulates noise to generate different types of pulsing dynamics
Source: PLoS Comput Biol. 2023 Aug 4;19(8):e1011265. doi: 10.1371/journal.pcbi.1011265 (PMC10431680; doi:10.1371/journal.pcbi.1011265)
Supplement: S17 Fig — In Fig 4 we observed that the system undergoes a behavioural transition as pprod is increased from small to high. For small values of pprod the system is inactive. As pprod is increased the system exhibits, in order, single response pulse, stochastic pulsing, oscillating, and persistent activity, behaviours. Here, we recreate the same transition using Gillespie simulations. While we in Fig 4 vary the parameter pprod, we never introduce this parameter substitution for the Gillespie approach. We instead vary the parameter pstress. However, since pprod = pstress ⋅ kP, the two transitions should be equivalent. (A-L) Gillespie simulation of the Narula model for various values of pstress. Each frame contains 4 simulations, and stress is added at the red dashed lines (t = 0). The behavioural transition from Fig 4 is recreated. Parameter values and other details on simulation conditions for this figure are described in S3 Table. (PDF) [file pcbi.1011265.s017.pdf]

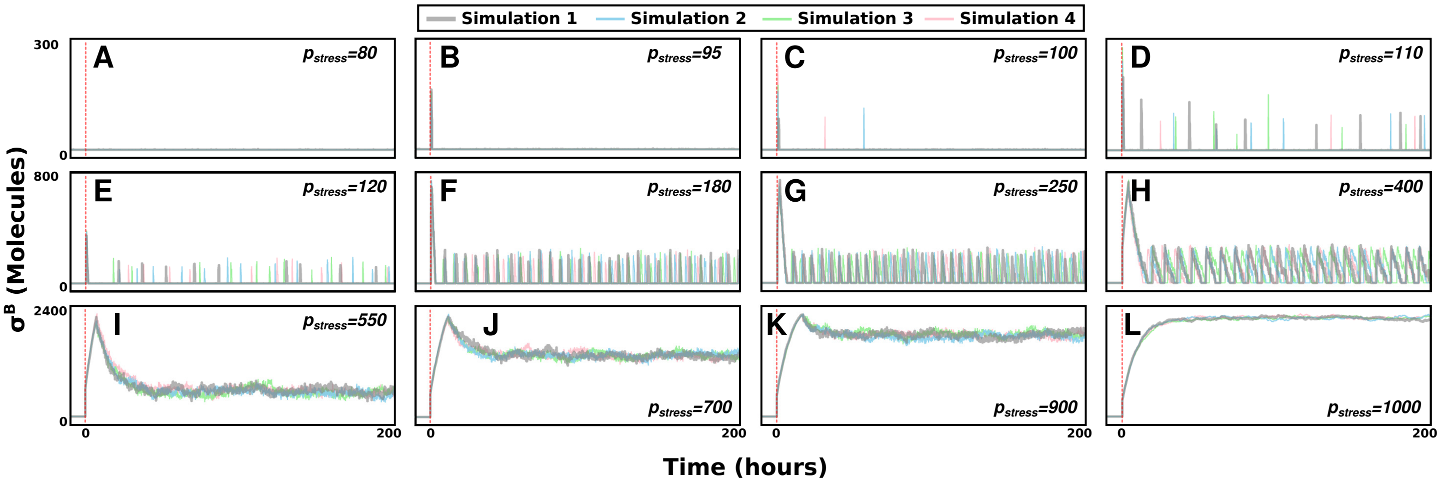

S Fig 17. The behavioural transition, as p_{prod} is varied, in Fig 4 can be recreated using the Gillespie algorithm. In Fig 4 we observed that the system undergoes a behavioural transition as p_{prod} is increased from small to high. For small values of p_{prod} the system is inactive. As p_{prod} is increased the system exhibits, in order, single response pulse, stochastic pulsing, oscillating, and persistent activity, behaviours. Here, we recreate the same transition using Gillespie simulations. While we in Fig 4 vary the parameter p_{prod}, we never introduce this parameter substitution for the Gillespie approach. We instead vary the parameter p_{stress}. However, since $p_{prod} = p_{stress} \cdot k_P$, the two transitions should be equivalent. (A-L) Gillespie simulation of the Narula model for various values of p_{stress}. Each frame contains 4 simulations, and stress is added at the red dashed lines ($t = 0$). The behavioural transition from Fig 4 is recreated. Parameter values and other details on simulation conditions for this figure are described in S3 Table.
