# Supplementary material for: The σB alternative sigma factor circuit modulates noise to generate different types of pulsing dynamics
Source: PLoS Comput Biol. 2023 Aug 4;19(8):e1011265. doi: 10.1371/journal.pcbi.1011265 (PMC10431680; doi:10.1371/journal.pcbi.1011265)
Supplement: S18 Fig — By splitting the phosphatase into an active state (P) and an inactive state (Pi), with only the active state being able to form the P-VP complex, we can introduce fluctuations in phosphatase levels. Before the addition of stress, the total amount of phosphatase ([Pi] + [P] + [P-VP]) is 2pinit (with, on average, at every time point, half being in the active state). At the time of stress onset (here at the red dashed line at t = 0), the amount of phosphatase is increased to 2pstress (again, with on average half being active). The frequency of the fluctuations scales with the parameter ηfreq. The amplitude of the fluctuations scales with the parameter ηamp. Parameter values and other details on simulation conditions for this figure are described in S7 Table. (PDF) [file pcbi.1011265.s018.pdf]

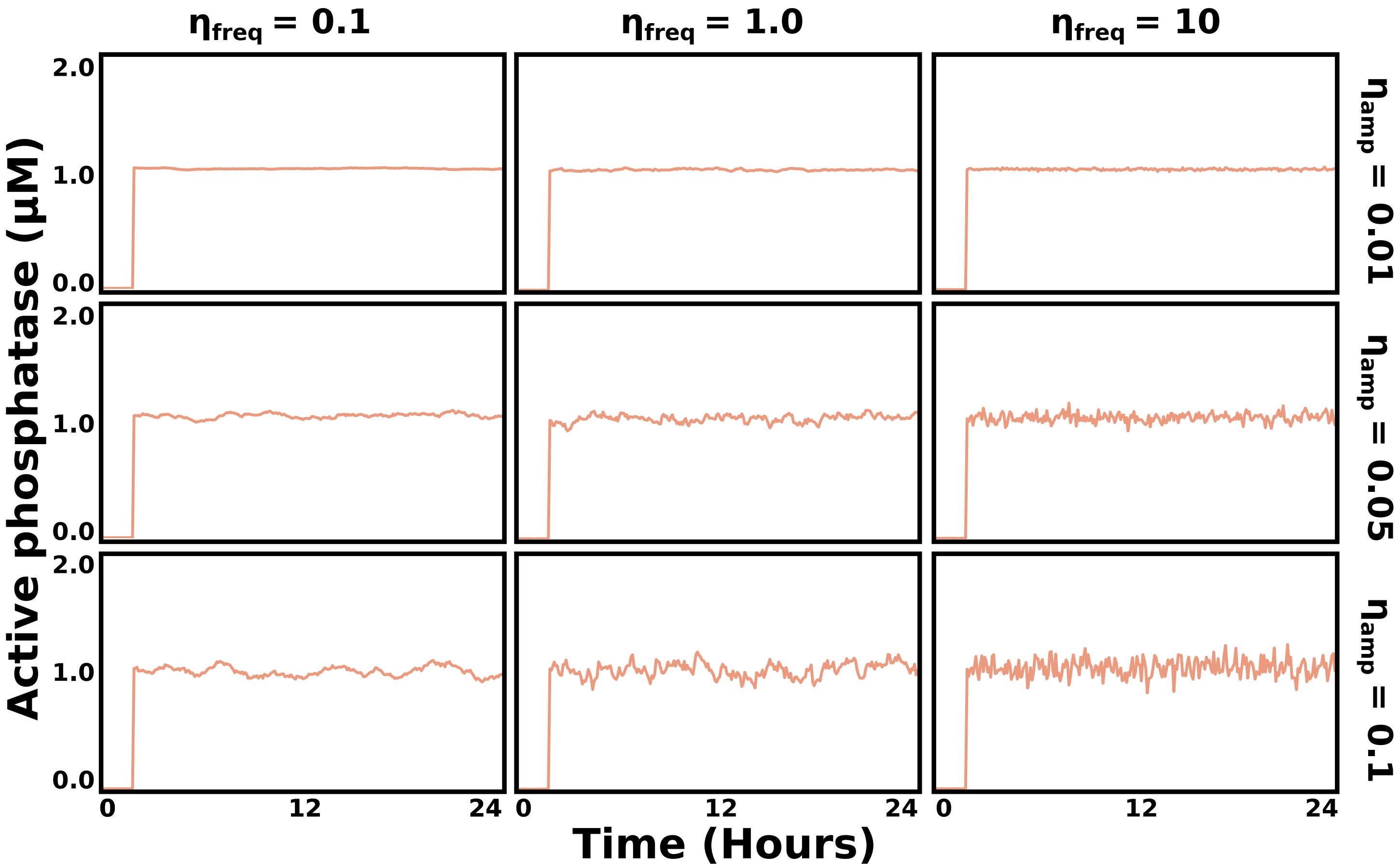

S Fig 18. In the modified Narula model, two parameters (η_{amp} and η_{freq}), allow us to scale the amplitude and frequency of the noise in the input phosphatase. By splitting the phosphatase into an active state (P) and an inactive state (P_i), with only the active state being able to form the P-VP complex, we can introduce fluctuations in phosphatase levels. Before the addition of stress, the total amount of phosphatase ($[P_i] + [P] + [P\text{-}VP]$) is $2p_{init}$ (with, on average, at every time point, half being in the active state). At the time of stress onset (here at the red dashed line at $t = 0$), the amount of phosphatase is increased to $2p_{stress}$ (again, with on average half being active). The frequency of the fluctuations scales with the parameter η_{freq}. The amplitude of the fluctuations scales with the parameter η_{amp}. Parameter values and other details on simulation conditions for this figure are described in S7 Table.
