# Supplementary material for: The σB alternative sigma factor circuit modulates noise to generate different types of pulsing dynamics
Source: PLoS Comput Biol. 2023 Aug 4;19(8):e1011265. doi: 10.1371/journal.pcbi.1011265 (PMC10431680; doi:10.1371/journal.pcbi.1011265)
Supplement: S1 Table — For each figure where the model is simulated, the parameter values used for the simulations are marked. If not marked, the following parameter values are used: kBw = 3600 μM-1hr-1, kDw = 18 hr-1, kB1 = 3600 μM-1hr-1, kB2 = 3600 μM-1hr-1, kB3 = 3600 μM-1hr-1, kB4 = 1800 μM-1hr-1, kB5 = 3600 μM-1hr-1, kD1 = 18 hr-1, kD2 = 18 hr-1, kD3 = 18 hr-1, kD4 = 1800 μM-1hr-1, kD5 = 18 hr-1, kK1 = 36 hr-1, kK2 = 36 hr-1, kP = 180 hr-1 hr-1, kDeg = 0.7 hr-1, v0 = 0.4 μM-1hr-1, F = 30, K = 0.2 μM, λW = 4, λV = 4.5, η = 0.05, pinit = 0.001 μM, pstress = 0.4 μM. Finally, in certain figures, some parameter values are varied as marked on the figures. Which parameters are varied across each is marked in the last column. (PDF) [file pcbi.1011265.s019.pdf]

Figure	Parameter values	Varied parameters
Fig 2A,B	$p_{init} = 0.001$ µM, $p_{stress} = 0.8$ µM	η
Fig 2C	$k_{K2} = 12.0$ hr^{-1}, $k_P = 320$ hr^{-1}, $p_{init} = 0.01$ µM, $\eta = 0.05$, $p_{stress} = 0.165$ µM	
Fig 2D	$k_{K2} = 12.0$ hr^{-1}, $k_P = 255$ hr^{-1}, $p_{init} = 0.2$ µM, $\eta = 0.05$, $p_{stress} = 0.23$ µM	
Fig 2G	$k_{K2} = 15.0$ hr^{-1}, $p_{init} = 0.05$ µM, $\eta = 0.04$, $p_{stress} = 0.4$ µM	
Fig 2H	$k_{K2} = 9.0$ hr^{-1}, $p_{init} = 0.05$ µM, $\eta = 0.06$, $p_{stress} = 0.225$ µM	
Sup Fig 1		p_{stress}, η
Sup Fig 2A,B		All parameters
Sup Fig 3A		k_{K2}, λ_V, p_{stress}
Sup Fig 5A,B	$k_{K2} = 9.0$ hr^{-1}, $p_{init} = 0.05$ µM, $\eta = 0.055$, $p_{stress} = 0.225$ µM	
Sup Fig 8A	$k_{K2} = 7.0$ hr^{-1}, $p_{init} = 0.05$ µM, $\eta = 0.025$, $p_{stress} = 0.24$ µM	
Sup Fig 8B	$k_{K2} = 7.0$ hr^{-1}, $p_{init} = 0.05$ µM, $\eta = 0.025$, $p_{stress} = 0.28$ µM	

S Table 1. Parameter values for simulation of the CLE adaptation of the Narula model. For each figure where the model is simulated, the parameter values used for the simulations are marked. If not marked, the following parameter values are used: $k_{Bw} = 3600$ µM^{-1}hr^{-1}, $k_{Dw} = 18$ hr^{-1}, $k_{B1} = 3600$ µM^{-1}hr^{-1}, $k_{B2} = 3600$ µM^{-1}hr^{-1}, $k_{B3} = 3600$ µM^{-1}hr^{-1}, $k_{B4} = 1800$ µM^{-1}hr^{-1}, $k_{B5} = 3600$ µM^{-1}hr^{-1}, $k_{D1} = 18$ hr^{-1}, $k_{D2} = 18$ hr^{-1}, $k_{D3} = 18$ hr^{-1}, $k_{D4} = 1800$ µM^{-1}hr^{-1}, $k_{D5} = 18$ hr^{-1}, $k_{K1} = 36$ hr^{-1}, $k_{K2} = 36$ hr^{-1}, $k_P = 180$ hr^{-1} hr^{-1}, $k_{Deg} = 0.7$ hr^{-1}, $v_0 = 0.4$ µM^{-1}hr^{-1}, $F = 30$, $K = 0.2$ µM, $\lambda_W = 4$, $\lambda_V = 4.5$, $\eta = 0.05$, $p_{init} = 0.001$ µM, $p_{stress} = 0.4$ µM. Finally, in certain figures, some parameter values are varied as marked on the figures. Which parameters are varied across each is marked in the last column.
