# Supplementary material for: The σB alternative sigma factor circuit modulates noise to generate different types of pulsing dynamics
Source: PLoS Comput Biol. 2023 Aug 4;19(8):e1011265. doi: 10.1371/journal.pcbi.1011265 (PMC10431680; doi:10.1371/journal.pcbi.1011265)
Supplement: S2 Table — For each figure where we perform parameter scans of the model’s behaviour, the parameter values used are marked. If not marked, the following parameter values are used: kBw = 3600 μM-1hr-1, kDw = 18 hr-1, kB1 = 3600 μM-1hr-1, kB2 = 3600 μM-1hr-1, kB3 = 3600 μM-1hr-1, kB4 = 1800 μM-1hr-1, kB5 = 3600 μM-1hr-1, kD1 = 18 hr-1, kD2 = 18 hr-1, kD3 = 18 hr-1, kD4 = 1800 μM-1hr-1, kD5 = 18 hr-1, kK1 = 36 hr-1, kK2 = 36 hr-1, kP = 180 hr-1, kDeg = 0.7 hr-1, v0 = 0.4 μM-1hr-1, F = 30, K = 0.2 μM, λW = 4, λV = 4.5, η = 0.05, and pinit = 0.001 μM. The third column denotes how many simulations (n) are performed for each parameter combination. Finally, in certain figures, some parameter values are varied as marked on the figures. Which parameters are varied across each is marked in the last column. (PDF) [file pcbi.1011265.s020.pdf]

Figure	Parameter values	Simulations (n)	Varied parameters
Fig 2E,F		150	p_{stress}, k_{K2}, η
Fig 3A,B	$k_{K2} = 7$ hr^{-1}, $\eta = 0.025$	100	k_P, p_{stress}
Sup Fig 6	$k_{K2} = 7$ hr^{-1}, $\eta = 0.025$	150	p_{stress}
Sup Fig 7		150	p_{stress}, k_{K2}, η
Sup Fig 9	$k_{K2} = 7$ hr^{-1}, $\eta = 0.025$	100	k_P, p_{stress}, $kB5$, k_{D5}

S Table 2. Parameter values for parameter scans of the CLE adaptation of the Narula model. For each figure where we perform parameter scans of the model's behaviour, the parameter values used are marked. If not marked, the following parameter values are used: $k_{Bw} = 3600$ µM^{-1}hr^{-1}, $k_{Dw} = 18$ hr^{-1}, $k_{B1} = 3600$ µM^{-1}hr^{-1}, $k_{B2} = 3600$ µM^{-1}hr^{-1}, $k_{B3} = 3600$ µM^{-1}hr^{-1}, $k_{B4} = 1800$ µM^{-1}hr^{-1}, $k_{B5} = 3600$ µM^{-1}hr^{-1}, $k_{D1} = 18$ hr^{-1}, $k_{D2} = 18$ hr^{-1}, $k_{D3} = 18$ hr^{-1}, $k_{D4} = 1800$ µM^{-1}hr^{-1}, $k_{D5} = 18$ hr^{-1}, $k_{K1} = 36$ hr^{-1}, $k_{K2} = 36$ hr^{-1}, $k_P = 180$ hr^{-1}, $k_{Deg} = 0.7$ hr^{-1}, $v_0 = 0.4$ µM^{-1}hr^{-1}, $F = 30$, $K = 0.2$ µM, $\lambda_W = 4$, $\lambda_V = 4.5$, $\eta = 0.05$, and $p_{init} = 0.001$ µM. The third column denotes how many simulations (n) are performed for each parameter combination. Finally, in certain figures, some parameter values are varied as marked on the figures. Which parameters are varied across each is marked in the last column.
