# Supplementary material for: The σB alternative sigma factor circuit modulates noise to generate different types of pulsing dynamics
Source: PLoS Comput Biol. 2023 Aug 4;19(8):e1011265. doi: 10.1371/journal.pcbi.1011265 (PMC10431680; doi:10.1371/journal.pcbi.1011265)
Supplement: S3 Table — Here, Ms stand for Molecules. For each figure where the Narula model is simulated using the Gillespie algorithm, the parameter values used for the simulations are marked. If not marked, the following parameter values are used: kBw = 3600 Molecules-1hr-1, kDw = 18 hr-1, kB1 = 3600 Molecules-1hr-1, kB2 = 3600 Molecules-1hr-1, kB3 = 3600 Molecules-1hr-1, kB4 = 1800 Molecules-1hr-1, kB5 = 3600 Molecules-1hr-1, kD1 = 18 Molecules-1, kD2 = 18 hr-1, kD3 = 18 hr-1, kD4 = 1800 Molecules-1hr-1, kD5 = 18 hr-1, kK1 = 36 hr-1, kP = 180 hr-1, λW = 4, λV = 4.5, and pinit = 0 Molecules. Finally, in S18 Fig, the value of pstress is varied as marked in the figure. This is designated by, in this table, putting pstress in the “Varied parameters” column. (PDF) [file pcbi.1011265.s021.pdf]

Figure	Parameter values	Varied parameters
Fig 2I	$k_{K2} = 18$ hr^{-1}, $k_{deg} = 0.1$ hr^{-1}, $v_0 = 1.0$ Ms·hr^{-1}, $F = 300$, $K = 0.02$ Ms, $p_{stress} = 58$ Ms	
Fig 2J	$k_{K2} = 18$ hr^{-1}, $k_{deg} = 0.1$ hr^{-1}, $v_0 = 1.6$ Ms·hr^{-1}, $F = 300$, $K = 0.02$ Ms, $p_{stress} = 115$ Ms	
Sup Fig 17	$k_{K2} = 18$ hr^{-1}, $k_{deg} = 0.1$ hr^{-1}, $v_0 = 1.6$ Ms·hr^{-1}, $F = 300$, $K = 0.02$ Ms	p_{stress}

S Table 3. Parameter values for Gillespie simulation of the Narula model. Here, Ms stand for Molecules. For each figure where the Narula model is simulated using the Gillespie algorithm, the parameter values used for the simulations are marked. If not marked, the following parameter values are used: $k_{Bw} = 3600$ Molecules^{-1}hr^{-1}, $k_{Dw} = 18$ hr^{-1}, $k_{B1} = 3600$ Molecules^{-1}hr^{-1}, $k_{B2} = 3600$ Molecules^{-1}hr^{-1}, $k_{B3} = 3600$ Molecules^{-1}hr^{-1}, $k_{B4} = 1800$ Molecules^{-1}hr^{-1}, $k_{B5} = 3600$ Molecules^{-1}hr^{-1}, $k_{D1} = 18$ Molecules^{-1}, $k_{D2} = 18$ hr^{-1}, $k_{D3} = 18$ hr^{-1}, $k_{D4} = 1800$ Molecules^{-1}hr^{-1}, $k_{D5} = 18$ hr^{-1}, $k_{K1} = 36$ hr^{-1}, $k_P = 180$ hr^{-1}, $\lambda_W = 4$, $\lambda_V = 4.5$, and $p_{init} = 0$ Molecules. Finally, in S17 Fig, the value of p_{stress} is varied as marked in the figure. This is designated by, in this table, putting p_{stress} in the "Varied parameters" column.
