# Supplementary material for: The σB alternative sigma factor circuit modulates noise to generate different types of pulsing dynamics
Source: PLoS Comput Biol. 2023 Aug 4;19(8):e1011265. doi: 10.1371/journal.pcbi.1011265 (PMC10431680; doi:10.1371/journal.pcbi.1011265)
Supplement: S4 Table — For each figure where the model is simulated, the parameter values used for the simulations are marked. If not marked, the following parameter values are used: kBw = 3600 μM-1hr-1, kDw = 18 hr-1, kB1 = 3600 μM-1hr-1, kB2 = 3600 μM-1hr-1, kB3 = 3600 μM-1hr-1, kB4 = 1800 μM-1hr-1, kB5 = 3600 μM-1hr-1, kD1 = 18 hr-1, kD2 = 18 hr-1, kD3 = 18 hr-1, kD4 = 1800 μM-1hr-1, kD5 = 18 hr-1, kK1 = 36 hr-1, kK2 = 36 hr-1, kDeg = 0.7 hr-1, v0 = 0.4 μM-1hr-1, F = 30, K = 0.2 μM, λW = 4, λV = 4.5, η = 0.05, pinit = 0.001 μM, and pfrac = 100 μM hr1. Finally, in certain figures, some parameter values are varied as marked on the figures. Which parameters are varied across each is marked in the last column. (PDF) [file pcbi.1011265.s022.pdf]

Figure	Parameter values	Varied parameters
Fig 4B-M	$k_{K2} = 7$ hr^{-1}, $\eta = 0.025$, $p_{frac} = 100$ µM hr^{1}	p_{prod}
Sup Fig 17B	$k_{K2} = 7$ hr^{-1}, $\eta = 0.025$, $p_{frac} = 100$ µM hr^{1}	p_{prod}
Sup Fig 17C-N	$k_{K2} = 7$ hr^{-1}, $\eta = 0.025$, $p_{frac} = 100$ µM hr^{1}	p_{prod}

S Table 4. Parameter values for simulation of the CLE adaptation of the Narula model with parameter substitution. For each figure where the model is simulated, the parameter values used for the simulations are marked. If not marked, the following parameter values are used: $k_{Bw} = 3600$ µM^{-1}hr^{-1}, $k_{Dw} = 18$ hr^{-1}, $k_{B1} = 3600$ µM^{-1}hr^{-1}, $k_{B2} = 3600$ µM^{-1}hr^{-1}, $k_{B3} = 3600$ µM^{-1}hr^{-1}, $k_{B4} = 1800$ µM^{-1}hr^{-1}, $k_{B5} = 3600$ µM^{-1}hr^{-1}, $k_{D1} = 18$ hr^{-1}, $k_{D2} = 18$ hr^{-1}, $k_{D3} = 18$ hr^{-1}, $k_{D4} = 1800$ µM^{-1}hr^{-1}, $k_{D5} = 18$ hr^{-1}, $k_{K1} = 36$ hr^{-1}, $k_{K2} = 36$ hr^{-1}, $k_{Deg} = 0.7$ hr^{-1}, $v_0 = 0.4$ µM^{-1}hr^{-1}, $F = 30$, $K = 0.2$ µM, $\lambda_W = 4$, $\lambda_V = 4.5$, $\eta = 0.05$, $p_{init} = 0.001$ µM, and $p_{frac} = 100$ µM hr^{1}. Finally, in certain figures, some parameter values are varied as marked on the figures. Which parameters are varied across each is marked in the last column.
