# Supplementary material for: The σB alternative sigma factor circuit modulates noise to generate different types of pulsing dynamics
Source: PLoS Comput Biol. 2023 Aug 4;19(8):e1011265. doi: 10.1371/journal.pcbi.1011265 (PMC10431680; doi:10.1371/journal.pcbi.1011265)
Supplement: S5 Table — For each figure where we perform parameter scans of the model’s behaviour, the parameter values used are marked. If not marked, the following parameter values are used: kBw = 3600 μM-1hr-1, kDw = 18 hr-1, kB1 = 3600 μM-1hr-1, kB2 = 3600 μM-1hr-1, kB3 = 3600 μM-1hr-1, kB4 = 1800 μM-1hr-1, kB5 = 3600 μM-1hr-1, kD1 = 18 hr-1, kD2 = 18 hr-1, kD3 = 18 hr-1, kD4 = 1800 μM-1hr-1, kD5 = 18 hr-1, kK1 = 36 hr-1, kK2 = 36 hr-1, kDeg = 0.7 hr-1, v0 = 0.4 μM-1hr-1, F = 30, K = 0.2 μM, λW = 4, λV = 4.5, η = 0.05, pinit = 0.001 μM, and pfrac = 100 μM hr1. The third column denotes how many simulations (n) are performed for each parameter combination. These simulations are then used to determine behavioural magnitudes (larger n yields smoother plots). Finally, in certain figures, some parameter values are varied as marked on the figures. Which parameters are varied across each is marked in the last column. (PDF) [file pcbi.1011265.s023.pdf]

Figure	Parameter values	Simulations (n)	Varied parameters
Fig 3C,D	$k_{K2} = 7$ hr^{-1}, $\eta = 0.025$	100	p_{prod}, p_{frac}
Fig 3E,F	$k_{K2} = 7$ hr^{-1}, $\eta = 0.025$	200	p_{prod}, p_{frac}, k_{B5}, k_{D5}
Fig 4A	$k_{K2} = 7$ hr^{-1}, $\eta = 0.025$, $p_{frac} = 100$ µM hr^{1}	200	p_{prod}
Sup Fig 10	$k_{K2} = 7$ hr^{-1}, $\eta = 0.025$	200	p_{prod}, p_{frac}, k_{B5}, k_{D5}
Sup Fig 11	$k_{K2} = 7$ hr^{-1}, $\eta = 0.025$	200	p_{prod}, p_{frac}, k_{B5}, k_{D5}
Sup Fig 12	$k_{K2} = 7$ hr^{-1}, $\eta = 0.025$	200	p_{prod}, p_{frac}, k_{B5}, k_{D5}
Sup Fig 13	$k_{K2} = 7$ hr^{-1}, $\eta = 0.025$	200	p_{prod}, p_{frac}, k_{B5}, k_{D5}
Sup Fig 14	$k_{K2} = 7$ hr^{-1}, $\eta = 0.025$	200	p_{prod}, p_{frac}, k_{B5}, k_{D5}
Sup Fig 15	$k_{K2} = 7$ hr^{-1}, $\eta = 0.025$	200	p_{prod}, p_{frac}, k_{B5}, k_{D5}
Sup Fig 16A	$k_{K2} = 7$ hr^{-1}, $\eta = 0.025$, $p_{frac} = 100$ µM hr^{1}	200	p_{prod}

S Table 5. Parameter values for parameter scans of the CLE adaptation of the Narula model with parameter substitution. For each figure where we perform parameter scans of the model's behaviour, the parameter values used are marked. If not marked, the following parameter values are used: $k_{Bw} = 3600$ µM^{-1}hr^{-1}, $k_{Dw} = 18$ hr^{-1}, $k_{B1} = 3600$ µM^{-1}hr^{-1}, $k_{B2} = 3600$ µM^{-1}hr^{-1}, $k_{B3} = 3600$ µM^{-1}hr^{-1}, $k_{B4} = 1800$ µM^{-1}hr^{-1}, $k_{B5} = 3600$ µM^{-1}hr^{-1}, $k_{D1} = 18$ hr^{-1}, $k_{D2} = 18$ hr^{-1}, $k_{D3} = 18$ hr^{-1}, $k_{D4} = 1800$ µM^{-1}hr^{-1}, $k_{D5} = 18$ hr^{-1}, $k_{K1} = 36$ hr^{-1}, $k_{K2} = 36$ hr^{-1}, $k_{Deg} = 0.7$ hr^{-1}, $v_0 = 0.4$ µM^{-1}hr^{-1}, $F = 30$, $K = 0.2$ µM, $\lambda_W = 4$, $\lambda_V = 4.5$, $\eta = 0.05$, $p_{init} = 0.001$ µM, and $p_{frac} = 100$ µM hr^{1}. The third column denotes how many simulations (n) are performed for each parameter combination. These simulations are then used to determine behavioural magnitudes (larger n yields smoother plots). Finally, in certain figures, some parameter values are varied as marked on the figures. Which parameters are varied across each is marked in the last column.
