# Supplementary material for: The σB alternative sigma factor circuit modulates noise to generate different types of pulsing dynamics
Source: PLoS Comput Biol. 2023 Aug 4;19(8):e1011265. doi: 10.1371/journal.pcbi.1011265 (PMC10431680; doi:10.1371/journal.pcbi.1011265)
Supplement: S7 Table — For each figure where we perform parameter scans of the modified Narula model’s behaviour, the parameter values used are marked. If not marked, the following parameter values are used: kBw = 3600 μM-1hr-1, kDw = 18 hr-1, kB1 = 3600 μM-1hr-1, kB2 = 3600 μM-1hr-1, kB3 = 3600 μM-1hr-1, kB4 = 1800 μM-1hr-1, kB5 = 3600 μM-1hr-1, kD1 = 18 hr-1, kD2 = 18 hr-1, kD3 = 18 hr-1, kD4 = 1800 μM-1hr-1, kD5 = 18 hr-1, kK1 = 36 hr-1, kK2 = 36 hr-1, kDeg = 0.7 hr-1, v0 = 0.4 μM-1hr-1, F = 30, K = 0.2 μM, λW = 4, λV = 4.5, η = 0.05, pinit = 0.001 μM, pfrac = 100 μM hr1, ηamp = 0.05, and ηfreq = 1. The third column denotes how many simulations (n) are performed for each parameter combination. These simulations are then used to determine behavioural magnitudes (larger n yields smoother plots). Finally, in certain figures, some parameter values are varied as marked on the figures. Which parameters are varied across each is marked in the last column. (PDF) [file pcbi.1011265.s025.pdf]

Figure	Parameter values	Simulations (n)	Varied parameters
Fig 5A,B	$k_{K2} = 7$ hr^{-1}, $\eta = 0.025$, $p_{frac} = 100$ µM hr^{1}	100	p_{prod}, η_{amp}, η_{freq}
Fig 5C	$k_{K2} = 7$ hr^{-1}, $\eta = 0.025$, $p_{frac} = 100$ µM hr^{1}, $\eta_{amp} = 0.01$, $\eta_{freq} = 100$	100	p_{prod}
Fig 5D	$k_{K2} = 7$ hr^{-1}, $\eta = 0.025$, $p_{frac} = 100$ µM hr^{1}, $\eta_{amp} = 0.18$, $\eta_{freq} = 0.25$	100	p_{prod}
Fig 6A,B	$k_{K2} = 7$ hr^{-1}, $p_{frac} = 100$ µM hr^{1}, $\eta_{freq} = 1.0$	100	p_{prod}, η, η_{amp}

S Table 7. Parameter values for parameter scans of the modified Narula model. For each figure where we perform parameter scans of the modified Narula model's behaviour, the parameter values used are marked. If not marked, the following parameter values are used: $k_{Bw} = 3600$ µM^{-1}hr^{-1}, $k_{Dw} = 18$ hr^{-1}, $k_{B1} = 3600$ µM^{-1}hr^{-1}, $k_{B2} = 3600$ µM^{-1}hr^{-1}, $k_{B3} = 3600$ µM^{-1}hr^{-1}, $k_{B4} = 1800$ µM^{-1}hr^{-1}, $k_{B5} = 3600$ µM^{-1}hr^{-1}, $k_{D1} = 18$ hr^{-1}, $k_{D2} = 18$ hr^{-1}, $k_{D3} = 18$ hr^{-1}, $k_{D4} = 1800$ µM^{-1}hr^{-1}, $k_{D5} = 18$ hr^{-1}, $k_{K1} = 36$ hr^{-1}, $k_{K2} = 36$ hr^{-1}, $k_{Deg} = 0.7$ hr^{-1}, $v_0 = 0.4$ µM^{-1}hr^{-1}, $F = 30$, $K = 0.2$ µM, $\lambda_W = 4$, $\lambda_V = 4.5$, $\eta = 0.05$, $p_{init} = 0.001$ µM, $p_{frac} = 100$ µM hr^{1}, $\eta_{amp} = 0.05$, and $\eta_{freq} = 1$. The third column denotes how many simulations (n) are performed for each parameter combination. These simulations are then used to determine behavioural magnitudes (larger n yields smoother plots). Finally, in certain figures, some parameter values are varied as marked on the figures. Which parameters are varied across each is marked in the last column.
