# Supplementary material for: The σB alternative sigma factor circuit modulates noise to generate different types of pulsing dynamics
Source: PLoS Comput Biol. 2023 Aug 4;19(8):e1011265. doi: 10.1371/journal.pcbi.1011265 (PMC10431680; doi:10.1371/journal.pcbi.1011265)
Supplement: Attachment — Submitted filename: Loman_Locke_2023_response_to_reviewers.pdf [file pcbi.1011265.s026.pdf]

We thank the reviewers for their careful reading of the manuscript and constructive comments. We have thoroughly revised our manuscript, adding multiple control simulations that were suggested by the reviewers. We have added supplementary figures 5, 17, 18, as well as Figure 2 panels G and H. Our replies are coloured purple. Line number references in our replies refer to line numbers in the PLOS formatted PDF file.

Reviewer 1

Summary

The paper by Loman and Locke presents a simulation-based study to understand how different types of pulsing dynamics may be modulated by noise in the σB alternative sigma factor circuit. They show that whether the response is stochastic pulsing or single response pulse is determined by the degree with which the input pathway activates the core circuit and also by the stochastic properties of the input pathway. The results are certainly interesting. However I have some concerns about the type of noise captured by their study (they ignore the major sources of extrinsic noise), and some details about the simulation methods appear missing.

Comments

1. In the Introduction they write "Traditionally such models have been deterministic in nature, however, due to low molecular copy numbers, cellular networks are often stochastic". I think this picture is now somewhat outdated. The noise they describe is intrinsic noise but many studies have shown that extrinsic sources of noise are at least equally important, if not more important than intrinsic noise. So for e.g. variation amongst cells due to cell size, doubling time, etc is a leading cause of why cellular dynamics is perceived to be stochastic. Indeed the literature in the past decade has moved towards incorporating explicitly various sources of extrinsic noise. A few papers investigating such sources:
    - Shahrezaei, Vahid, Julien F. Ollivier, and Peter S. Swain. "Colored extrinsic fluctuations and stochastic gene expression." Molecular systems biology 4.1 (2008): 196.
    - Benzinger, Dirk, and Mustafa Khammash. "Pulsatile inputs achieve tunable attenuation of gene expression variability and graded multi-gene regulation." Nature communications 9.1 (2018): 1-10.

- Perez-Carrasco et al. "Effects of cell cycle variability on lineage and population measurements of messenger RNA abundance." Journal of the Royal Society Interface 17.168 (2020): 20200360.
- Ietswaart, Robert, et al. "Cell-size-dependent transcription of FLC and its antisense long non-coding RNA COOLAIR explain cell-to-cell expression variation." Cell Systems 4.6 (2017): 622-635.

Reply: We agree that the introduction sounded outdated and did not represent the field accurately. We have rewritten the introduction to reflect more accurately the state of the art of the field and to describe the different possible sources of noise. We have also included the references suggested by the reviewer. Please see lines 8-15.

2. The present study is based on the use of the chemical Langevin equation which has a system size parameter in it -- they consider this to be a parameter that scales the amplitude of the noise in the system but actually the system size is the cell volume (see van Kampen's book) and hence this varies from cell to cell. The experimental confirmation of the predictions they are making will necessarily involve a population of cells with an associated cell size distribution and even if one can follow a single cell, its size would vary with time. Its hence unclear to me how how much the addition of these relevant types of extrinsic noise will impact the present results. I suggest they modify their SDE approach to account for some of the relevant sources e.g. cell size variation in a population.

   Reply: We agree with the reviewer that multiple sources of noise are possible in the σ^B circuit, including both intrinsic and extrinsic factors. We have now added extra reasoning for why we feel it is reasonable to not consider cell size variation in a population in this model, and to focus on intrinsic noise (lines 116-121). Current experimental evidence (Locke et al., Science 2011, Park et al., 2018) points to transcriptional noise driving alternative sigma factor pulse initiation under energy stress and not extrinsic factors such as the cell cycle. There are no correlations between sister cell pulsing or between mother cells and daughter cells. Of course, this does not completely rule out the effects of cell size on pulsing, and we now discuss this limitation in the discussion and how we should consider other sources of noise in both experiments and modelling in future work (lines 332-337). As discussed below, we now reproduce our key results with simulations using the Gillespie algorithm, which we hope re-assures the reviewer that our assumptions and conclusions are solid and not dependent on the assumptions of the CLE.

3. It should be explicitly mentioned that the CLE is a valid approach in the limit of small noise (I could not find this mentioned). It is known that if the CLE simulations approach zero molecule numbers then there is a possibility that the

numbers go negative which is unrealistic. Often an artificial reflective boundary is thus implemented such that if the molecule number goes negative then it is set back to zero. This can lead to artifactual effects however in the simulation output. Have the authors of the present study employed such a reflective boundary?

Reply: As suggested by the reviewer, we now discuss in more detail the limitations of the CLE approach in the introduction, and point out our use of the reflective boundary and that the CLE is a valid approach in the limit of small noise (lines 105-107, 113-114, 311-320). We believe that the concerns about our use of the CLE should now be alleviated as we have produced additional figures (Figure 2G,H and Supplementary Figure 18) using the Gillespie algorithm that reproduces the key findings of our study.

4. According to Table 1, dilution is modelled via effective first-order reactions. While this is commonly done, it has in recent years been shown to be inaccurate when compared to a model which explicitly models dilution, i.e. models cell division and the partitioning of molecules between daughter cells. See for e.g. Beentjes, et al. "Exact solution of stochastic gene expression models with bursting, cell cycle and replication dynamics." Physical Review E 101.3 (2020): 032403. I am not suggesting that the authors modify their model to take into account this more realistically but at least this should be adequately discussed as a limitation of the present model emphasizing that effective dilution reactions underestimate the true size of the noise from partitioning at cell division; as well, some discussion should be added on how this might affect the present results.

   Reply: Thank you for this interesting and important point. We now discuss this limitation of our approach and how it could affect our predictions in the discussion. We have also referenced the paper suggested by the reviewer (lines 334-337).

Typos

1. On P. 13 in the equation for [W_2] there is a missing bracket in the fourth term on the RHS.
2. On P. 8 "simualte" should be "simulate".

   Reply: Fixed, thank you!

Reviewer 2

Summary

In this manuscript the authors analyse a model of bacterial stress response in the presence of noise. In particular, the authors show that a variant of a previously published model of the alternative sigma factor SigB response can generate two different responses (single pulse response vs stochastic pulsing) depending on the input stress.

Their main finding is that their stochastic model can give rise to either type of response for deterministic stress input. This extends the results reported previously in the literature which analysed a deterministic model which could only generate single pulse responses when subjected to deterministic step-change in input stress.

Bacterial stress response and the effect of noise on biochemical reaction pathways is an important area of much recent experimental and theoretical interest. However, I have several misgivings about the presented research.

Comments

1. It is unclear what the wider scientific contribution to the field is. Before the authors' current work, the field had analysed the deterministic version of their model which generated stochastic responses only when the input was stochastic. The fact that a deterministic model can only give deterministic outputs to deterministic inputs is of course a given. In the current manuscript the authors added some noise to the system and showed that now even a deterministic input can give rise to stochastic output behaviour. While it is a rewarding exercise to show this explicitly, I am sceptical this result significantly contributes to the field because the result comes as no surprise and is expected behaviour. In addition, even on a technical level it is unclear to me that a significant technical contribution was made. That is because of the way the authors add noise to the system as detailed next.

   Reply: We thank the reviewer for their thorough and insightful review of our work. They point out several areas where our analysis and reporting should be improved, which we hope they agree that we have implemented in this revised text. In terms of the first major point, that it is not clear how our model goes beyond previous results in the field, we would like to clarify what we consider the most novel aspects of our work. Our analysis showing that the core σ^B circuit is capable of generating both types of output dynamics goes beyond previous models and helps explain conflicting results in the field. It is also unexpected that simply raising phosphatase levels can result in a switch in behaviour from single pulse dynamics to stochastic pulsing. Our results motivate new experimental tests of the σ^B circuit. More generally, our work gives an example of how gene

circuits can modulate intrinsic noise to generate a range of output dynamics. Our analysis of the noise in the system goes beyond previous models and helps our understanding of stochastic pulsing in sigma factor circuits, which appears to be a general phenomenon. We believe our results should be of general interest, and we note that reviewers 1 and 3 share this opinion.

The second major aspects of the reviewer's comments are with our choice of the CLE approach (these concerns were also shared with reviewer 1). We believe that we have dealt with these concerns in this revision by recapitulating the key results of our study using the Gillespie algorithm, as well as adding extra simulations with the CLE as requested by the reviewer. We will discuss these results in detail below in response to the reviewer's specific points.

2. The authors add noise by analysing Langevin type dynamics with a global ad hoc noise strength parameter that affects all components. This seems like an unrealistic/unreliable approach to elucidate the actual effect of stochastic noise in the biochemical response dynamics. The authors comment on this very briefly in their introduction and support their choice by an argument of convenience. I find the lack of scientific justification unconvincing. Because the author's approach unavoidably randomizes the immediate effect of the input stress in the signal, I cannot see how the authors exclude the possibility that their observed dynamics is essentially equivalent to the previously observed one: a deterministic input that is immediately randomized in the first signalling event is essentially equivalent to adding noise to the input in the first place (which had been analysed previously). All the noise added to the remaining steps might not have played any role.

   Reply: Thank you for raising the issue of the use of the Chemical Langevin approach. We have now expanded our discussion on the CLE vs Gillespie approaches, describing the advantages and disadvantages of each in more detail (lines 98-121). While the Gillespie algorithm is the most exact approach, the CLE is a well established approximation, shown to be more exact than other alternatives such as the linear noise approximation. Indeed, CLE simulations have often been used to model gene regulatory networks when Gillespie simulations are intractable due to long run times (e.g Mavroudis et al. *Physiol Genomics* 2012, Perez-Carrasco et al. *PLoS Computational Biology* 2016, Manning et al. *Nature Communications* 2019, Perez-Carrasco et al. *Cell Systems* 2018), such as in our case for the parameter scans. To alleviate concerns about our use of the CLE and to improve the generality of our conclusions, we now reproduce key results using the Gillespie algorithm (which does not depend on a noise parameter eta). These new simulations are described below in reply to other specific comments from the reviewer.

With regard to the second comment. We now generate a modified model (simulated with the CLE approach) where we remove all forms of noise in reactions involving the Phosphatase P, and show that we can still recreate both single pulse and stochastic pulsing dynamic behaviours (Supplementary Figure 5). This further demonstrates that both behaviours can be reproduced from core noise only, without any upstream noise.

3. The work does not take into account that multiple sigma factors compete for a limited pool of RNA polymerases. Such competition effects have been shown to play an important role in bacterial stress response. In fact, one of the main points of the previous analysis of the deterministic model was to determine the effect of competition. Since the authors base their work on this previous model, they ought to include the same type of analysis.

   Reply: We agree with the reviewer that examining the effect of sigma factor competition on the dynamics of σ^B is an interesting analysis. We have implemented competition in the model (following the implementation of competition between σ^B and σ^A developed in the Narula model paper) and find that we are still able to generate stochastic pulsing and single pulse dynamics. However, a full analysis of this phenomenon is beyond the scope of this paper and we decided not to include this figure (which we, for the benefit of the reviewer, provide here). We do now discuss the possibility of such analysis as future work in the discussion section of the paper (line 343-345).

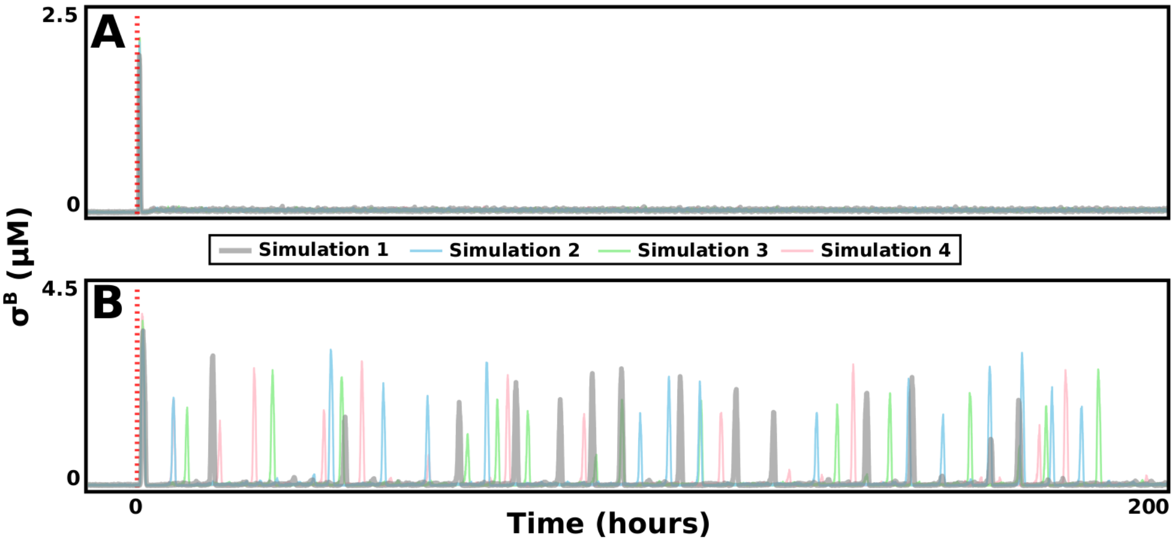

Both behaviours can be reproduced in a model where σ^B faces competition for RNA Polymerase from another sigma factor. In Narula (2016) they investigated the effect on the σ^B circuit when σ^B faced competition for RNA Polymerase from an additional sigma factor σ^A, showing that the behaviour of the circuit was robust to such competition. This is an implementation of the competition model from Narula (2016), with noise introduced via the CLE with a noise scaling parameter η. We show that both the single response pulse (A, $k_{K2}=15$, $p_{stress}=0.4$, $\eta=0.04$) and stochastic pulsing behaviours (B, $k_{K2}=9$, $p_{stress}=0.16$, $\eta=0.06$) can be recreated even as RNA Polymerase competition is taken into account. Stress is added at red dashed lines ($t=0$) and each plot shows four simulations. For both A and B, the amount of σ^B is set to $12\ \mu M$, defined in the Narula paper as the "high-competition" regime.

4. The manuscript is often unclear on crucial technical details to the point that the overall presentation is too imprecise for a scientific publication. I will detail some of these issues explicitly below as well as some of the minor typographic/presentation errors I noticed throughout.

   Reply: We thank the reviewer for their careful reading of the manuscript and we apologise for not spotting the typographic errors before submission. We outline below our improvements and hope that the reviewer agrees that the overall presentation is now precise enough for publication.

5. L246 The authors write "We use a (slightly altered) implementation of the core SigB circuit model implemented in [41]" Exactly how is this model altered? There is a sentence that follows explaining the model, but it only talks about the difference that P is a species. Is that all? Please clarify explicitly and explain in words how the model is different.

   Reply: We now describe our "alteration" directly in the text so as not to cause confusion (lines 360-375). Basically, in the Narula model P (as a species) was initialised at a starting concentration, and then at the time of stress increased to a different concentration. What we did was simply to formalise these two P concentrations (before and after stress) as parameters of the model, which essentially are what they are.

   This should also explain the confusion concerning whether P is a parameter or a species. Indeed, it would be possible to create a model where P was modelled as a parameter only, however, we decided against making non-needed changes to the Narula model.

   If P is now a species in their model, I did not understand its dynamics. The authors write "The strength of the stress is determined by the concentration of the species P." How is P increased? Should be specified/explained. This was confusing because reading through the results it seemed like P was *not* a species but just an input parameter that was instantaneously changes.

Reply: See previous response, P is a species and its pre/post stress concentration depends on two different parameters. We agree that the previous phrasing was confusing, and hence we have improved our description of the model.

As is, the presentation was very confusing, for example, the authors write "We will instead perform CLE based SDE simulations of the model. All inputs will be step increases in [P]." Which seems to contradict the model definition which suggests that noise was added to all species. Was P not a model species despite the statement that their model “models the input phosphatase, P, as a species of the system” or was it a species that was not subject to noise? While I am sure the authors are entirely clear in the way they set up their model the way it is described in this manuscript is not precise.

Reply: We thank the reviewer for pointing out this source of confusion. The CLE adds noise to the reactions of the system, not to the species themselves (of course, noise in reactions then ensures that species concentrations are noisy). Since P is neither produced nor degraded, the total amount of P will remain fixed (however, due to noise in reactions, the distribution of P across its various forms will vary). To make this clearer, we have improved our descriptions of the model (lines 360-375).

6. L266 The authors introduce a global noise parameter that affects the dynamics of all species. Later they vary this noise parameter, for example in Fig 1 eta ranges from 0 to: 0 0.15. It is unclear what this magnitude corresponds to in biochemical terms. Is 0.1 small or large?

   Reply: We set the range between 0 and 0.15 as this allows us to scan the parameter range between no noise (0) and high enough noise that it obscures most behaviours of the system (0.15). We now explain this in the text (SFig 8 caption).

7. L277 The authors write "In this section, we expand the Narula model, adding fluctuations to the upstream phosphatase input." I am confused by this statement as the Narula et al. work specifically considered noisy fluctuations in phosphatase levels.

   Reply: We agree that this sentence is confusing and so have re-written this in the text (lines 400-407).

8. L84 "To implement a stochastic CRN, we used the chemical Langevin equation (CLE) [42] We noted [sic] that it is also possible to simulate the CRN using the Gillespie algorithm [43, 44]. However, this interpretation does not allow for tuning of the system's degree of noise independently of its other properties."

   This is an argument based on convenience instead of scientific merit. This modelling choice is potentially problematic and should be discussed in detail. In fact, the authors included a supplementary figure (S1) that seems to show actual Gillespie simulations of a stochastic version of their system. I could not find any explanation of this figure/dynamics in the main results. Remarkably, the stochastic realizations of the Gillespie simulations did not seem exhibit any stochastic pulsing! This does not seem to be analysed or discussed anywhere in the paper.

   It is very odd for a supplementary figure like this to appear in a submitted manuscript. The only reference I could find to this simulation was in the methods "For the discrete simulations, we used Gillespie's direct stochastic simulation algorithm" but I could not find any mention of the results in the main text. The potential difference between the two types of dynamics is potentially a major scientific issue with the current analysis. It is of utmost importance to clarify for which part (if any) of the results discrete Gillespie simulations were run/relevant. Note, the supplementary figure S1 did not specify any of the parameter values apart from pinit=0. (Incidentally, why was pinit=0 chosen here when pinit=0.001 was specified in the parameter table?) It also seems the Gillespie simulations were done in an unrealistic parameter regime for just a handful of phosphatase molecules. Taken at face value the plots would suggest that the authors' main conclusions in the main text are misleading. This highlights a serious scientific issue with the current manuscript.

   Reply: We agree with the reviewer that the original simulations of the Gillespie algorithm are confusing, and due to this have removed supplemental figure 1 and replaced it with new simulations with the Gillespie algorithm that recapitulate our results using the CLE.

   Originally we kept all parameters the same as the original Narula model for the Gillespie simulation, to demonstrate that a Gillespie simulation of this parameter set can not produce stochastic pulsing, just as is the case for the CLE. We now simulate the Gillespie with an altered parameter set and demonstrate that the core circuit can generate both single pulse and stochastic pulsing behaviours (Fig 2G and H). In addition, we now show that we transition from single pulse, to

stochastic pulsing, to oscillations as we vary phosphatase levels in Gillespie simulations (Supplemental Figure 18), just as we do with the CLE.

We recognise that the Gillespie algorithm is desirable as it allows an exact simulation of the molecular reactions of the network. However, the approximations of the CLE enable quicker simulation, and this speed is essential for our analysis. The CLE has also commonly been used to model noise in biological networks, revealing insights into the behaviour of genetic circuits. The use of different approaches with various exactness depending on what is possible is common throughout science. However, we recognise that we were not clear about the advantages of the various approaches. We now discuss the CLE and Gillespie approaches more thoroughly (lines 98-121). We describe their advantages and disadvantages, and explain why we chose our approach. Finally, as mentioned above, the fact that we have reproduced key results using the Gillespie algorithm demonstrates that our results do not depend on the CLE (Figure 2 G,H, Supplementary Figure 18).

On the reviewer's point concerning initial conditions, the reviewer is correct that some simulations used *pinit=0.0* and some *pinit=0.001*, the difference is minuscule and has no impact on the simulations. Indeed, this likely only caused confusion and we now use *pinit=0.001* in the cases where *pinit=0.0* was used previously. We now provide tables with all parameter values of all simulations, hopefully, this should make it much easier to see the parameter values used for a single simulation.

9. Overall presentation is exceedingly difficult to follow. The key assumptions and which type of model was used for which kind of result should be made much clearer. The actual numerical details of the methods are crucial but either demoted to the methods or not even mentioned. Some key aspects should be folded into the main results text so it is possible to understand what quantity of which model under which perturbation one is looking at.

   For example, the distinction between single pulse and stochastic pulse dynamics is absolutely crucial for all their results. It should be explained as part of the results and not just in the methods. In fact, looking at the methods section it looks like the distinction is only made based on “time”, i.e., a single deterministic pulse is a response that is early, and everything that is late is classified as stochastic pulsing? If true, this should be highlighted. Also, the authors would need to show that this time-scale classification actually works to distinguish the two behaviours in *all* parameter regimes.

Note, that even after careful reading I do not fully understand the definitions of the responses. In line 328 the authors define the crucial measure as “we measure the magnitude of the single response pulse behaviour as the mean transient pulse amplitude divided by the mean asymptotic pulse amplitude”. However, the preceding bullet points do not define the “mean asymptotic pulse amplitude” but the "maximum asymptotic pulse amplitude" and "mean activity in the asymptotic state".

It is thus unclear what the term “mean asymptotic pulse amplitude” refers to. Imprecisions like this in language/presentations are extremely problematic and preclude the manuscripts’ results to contribute to the scientific literature.

Reply: We thank the reviewer for pointing out some of the difficulties they experienced in following the manuscript. In response, we have rewritten large parts of it, especially those parts that the reviewer found difficult. We hope that the new version will be easier for readers to follow.

This now includes an extensive description of our behavioural magnitude measures directly as they are introduced (Section 2.1, with further description in method sections 4.5.1). We also clarify that the measures are designed so that a parameter set exhibits either behaviour (e.g. a single pulse response behaviour requires the system not to exhibit stochastic pulses in the asymptotic phase, else it becomes stochastic pulsing).

Finally, we note that we set the transient phase to be 5 hours as this is much longer than the duration of a single pulse, which is typically of duration ~1 hour. To demonstrate that this is the case, we now perform a large scan of 10,000+ randomised parameter sets, ensuring that none have a transient phase > 5 hours (See Section 4.5.1).

10. Line 113 The authors claim "Through sample simulations, we demonstrated that the core circuit can generate both response behaviours, without making any assumptions about the upstream pathways (Figure 2D,E)." I disagree with the statement that no assumptions about the upstream pathways were made because the whole plot is for a *specific* type of upstream dynamics. I might be confused, but it doesn’t look like the authors meant what they stated here.

    Reply: We agree that our phrasing was confusing so have re-written this sentence. We meant that as we implemented the noise through the CLE for all system components, we did not make any different assumptions about the noise levels in the upstream components compared to the rest of the circuit. This contrasts with previous models, where a noisy input process was specified in the

upstream components. In our rewritten sentence we no longer suggest we make no assumptions (See lines 156-159).

11. Line 101 "Tuning of a combination of parameters can also induce pulsing. However, we wished to minimise the number of model parameter values we modified. The main reason for this is that the original parameter set was picked to be biologically plausible [41], making minimal deviations from this set desired. In addition, modifying a smaller set of parameters reduces the dimensionality of the parameter space we need to explore, reducing computational complexity. We thus selected k_{K2} only as our proxy for the system's proneness to pulsing."

    The argument to focus on just one parameter to avoid the complexity of the system's behaviour seems like an argument based on convenience rather than science.

    Making minimal deviations from the biologically plausible regime is sensible indeed, but that does not constrain the *number* of parameters changed. It constrains *how much* parameters should be changed. Having said that, I have no good intuition for how constrained the plausible range of the parameters presented in Table 2 really is. Are changes plus/minus a factor of two or a factor of 10 still plausible? I'd expect so, but the authors should clarify. For example, when the authors change k_{K2} the seem to deviate from the stated value of 36/h by a factor of 10. Why would it not be biologically plausible to change any of the other parameters similarly? Also, k_{K2} was changed only in one direction. Is it necessary for k_{K2} to be less than the plausible value?. I did not find a discussion of this anywhere.

    Reply: The plausibility of making a certain magnitude of change to a parameter depends on the parameter. Given the uncertainty of the initial guess, a factor of 10 should be well within the realm of plausibility. The reviewer states "*For example, when the authors change k_{K2} the seem to deviate from the stated value of 36/h by a factor of 10. Why would it not be biologically plausible to change any of the other parameters similarly?*". This is not the case, we test *all* parameters when deviated with a factor of 10 (typically reaching saturation of their effect on the system in each case). We then note that only two parameters allow oscillations under this regime. Due to k_{K2} requiring less of a change, and being the parameter for which this change seems more natural, we proceed with this only.

    As stated, we do agree that making a combination of changes to say 4 different parameters could likely induce oscillations. However, as both the reviewer and we point out, this greatly reduces the tractability of all following analyses. In this

case, what is important is that we have a way to tune the system's proneness to oscillations, whether from a single, or a set of, parameters. Indeed, if we had found a set of parameters able to induce oscillations when tuned together, we would next have performed parameter substitution to enable us to tune them "as one". This would have taken us back to where we currently are (tuning as a single parameter to induce oscillations), but in a much more convoluted way.

Finally, we do change k_{K2} in both directions (increase/decrease) by a factor 10 in our bifurcation diagrams (Supplementary Figure 2). As shown there, only a decrease in k_{K2} induces oscillation. We have made some changes to our bifurcation analysis section to improve clarity, and we think it should now be clearer what we do and why (lines 127-136).

12. Line 320 The authors state "To determine the magnitude of the behaviours for a given parameter set p… we simulate the system n times (n = 50, 100, or 200)" This is confusing. Which n is it?

    Reply: We agree that this sentence was confusing. When we, for a given parameter set, determine which type of behaviour it has, we simulate it n times and use some sample statistics to measure the degree of both behaviours. This n is the number of such simulations. We have now ensured that this is clearly described (See lines 150-152). Also, Supplementary tables 2, 5, and 7 contain all such *n* values for all parameter scans, ensuring that these can be easily found.

Figure Comments

There is a lack of detail and precision throughout the results figures. To pick out just a few that stood out the most to me:

Figure 2:

1. Panel B has response the scale missing. What is the intuitive explanation for the increase in amplitude with noise when all other parameters are equal? That seems not intuitive at all.

   Reply: The y-axis in B is the same as in A. We agree that this should be marked out and have now added it.

   When noise is added to a non-linear system, the effect often goes beyond simply noise around a mean. E.g. it is known that the mean concentration at a steady

state can increase with noise amplitude. The phenomenon that we see here is the same. While 2A and B are not a core part of the paper, this phenomenon is not as widely known as it could be. When we had an example of it in a real system we thought it would be useful for parts of our audience to be made aware that these kinds of effects exist.

2. The noise parameter is specified, but what are the other parameters? Are those the ones specified in Table 2? In particular, what was k_{K2} for the simulations in panel A,B?

   Reply: The Parameter values used have been the default values given in the methods, with the modifications described in figure legends. However, we recognise that these sometimes have been inaccessible (especially in this case where we made a reference to somewhere else in the text, which we agree was lazy). We have now compiled 7 supplementary tables, including all information on parameter values used throughout all figures.

3. Are the response amplitudes in panel A/B biologically realistic? If so, what about the response amplitudes in panels C/D which seem to be up to two orders of magnitude higher!

   Reply: This is a misunderstanding. E and F show the concentration of σ^B in the system. C and D show the magnitude of the two behaviours. These are defined as the maximum σ^B pulse amplitude (either in the transient or the asymptotic state), divided by some value, and hence take a very different range of values.

4. In Table 2 eta = 0.05 is specified. Where does that number come from, what does it mean intuitively, and is it biologically plausible?

   Reply: We would be happy to provide more details about eta. First we note that scaling the noise in the CLE is an established approach. We now include example references where the CLE has been implemented to examine gene regulatory network dynamics (refs 46-49). Within these references there are examples where equivalent formulations can be found, in which the CLE contains a parameter that relates to system size, and therefore, to noise intensity (e.g refs 47 and 49). Further examples include doi:10.1371/journal.pone.0073487, doi:10.1371/journal.pone.0031407, https://doi.org/10.1101/2022.12.27.521688.

   We note that, in practice, η is not a pure parameter of the system, but rather a composite of a large number of parameters (making our parameter set over-determined in the same way as a model where a rate is defined as k1*k2, which could be replaced by k without loss of generality). Hence, giving an estimate of this number is very challenging. Because of this fact, η takes a variety of values throughout the paper, and in Table 2 we just selected a representative

typical value. We now use 0.025 as this is the one most commonly used throughout the paper. However, we also note more clearly now that parameter values are changed throughout the paper, and point to where these can be found (supplementary tables 1-7).

5. Panel C/D is about the difference in single response pulse magnitude (SRPM) vs the stochastic pulsing amplitude (SPA) neither were defined at this point. There is not even a reference to the methods where the measures are defined. Only a reference to the supplementary figure S5. But this definition is key to everything that follows and cannot just be explained by a reference to a supplementary figure.

   Reply: We agree it makes sense to define these properly first, which we now do.

6. The result depicts the maximum SPA vs SRPM that can be achieved by changing p_stress. How exactly was this done, e.g., what is the range over which p_stress was varied? How did p_stress differ that maximizes the two different responses?

   Reply: p_{stress} was varied across the interval *20-200* and the maximum behaviour magnitude was chosen. While a larger range could be scanned, we always found the max within this range, so scanning a larger subset of (0,inf) was not required.

7. Panel E/F is described as depicting simulations that "maximise the pulses". But it is unclear what parameters were varied to maximise the response. If I had to guess, specific eta and k_{K2} were chosen. What were those, and what is the p_stress that corresponded to those simulations?

   Reply: As previously stated, we now ensure it is clear which parameter values are used in each simulation.

8. What are the units of p_stress and k_{K2}? Presumably, they are micromole and 1/hour (as in Table 2) but in the main text the units seem to have been casually dropped throughout.

   Reply: The units for each parameter are given in the methods. We have now also added these into the main text to avoid confusion.

9. The conclusion of Fig 2 reads "This demonstrates that the CRN of the Narula model can generate both behaviours, when exposed to intrinsic noise only." I disagree that this model is one that is exposed to intrinsic noise only. Because of the global noise parameter that affects all intrinsic components it seems that

some of the noise effect is due to what would be commonly called “extrinsic” sources.

Reply: Apologies for the confusion. Here, we were trying to differentiate between previous noise addition in the phosphatase levels in the Narula model versus the noise intrinsic to the core circuit here. It was a poor choice of words given the definitions of intrinsic/extrinsic noise and so we have rewritten this sentence (Figure 2 caption).

Figure 3

1. Panel A/B What are the parameter values chosen, for example p_init, but also all others? The results here confused me because it seems like the model ever only leads to significant stress responses for very specific values of the product P_prod = kp*P_stress. Taken at face value that would suggest that a fine-tuning of kp to the stress-level would be necessary for the response module to function properly. That seems to imply that the model does not behave like the real system? Alternatively, it is possible that my interpretation here is due to the graphical scale chosen. In essence, everything here looks black which might possibly include a lot of responses like the ones depicted in Fig 2? The graphical presentation is thus problematic because it is not possible to infer the response behaviour.

   Reply: Which parameter values are chosen should now be made more clear. Indeed, the range is rather slim. However, the relationship between the amount of stress and the amount of phosphatase (p_{stress}) in the system is unknown. Hence, while on the scale we have plotted the region of the response behaviour seems slim, this might not be the case in experiments.

   In the paper, we also note this phenomenon (lines 231-234). Indeed, we then go on to investigate what affects the robustness of the region in which either behaviour occurs. In Figure 5, we show that these regions can be made more robust by altering the amount of upstream noise. It is likely that other properties of the upstream pathways also help make the response robust.

2. What is the meaning of the parameter regime with significant response in SRPM and SPA? Looks like kp*P_stress~50 would lead to both responses. Which seems odd because the system would exhibit identical responses when all parameters are fixed. Does that mean the SRPM and SPA are non-exclusive or that the system randomly shows either behaviour? What are the implications for the interpretation of SRPM and SPA? What are experimental estimates for P_prod?

   Reply: The responses are exclusive in this region and do not overlap. The regions are adjacent to each other, and due to the scale in especially A, B it is hard to

distinguish (although the green lines help). See e.g. Figure 4A for how these two regions overlap only a bit at the edges, as the system transitions from one behaviour to the other.

3. Panel C/D what are the values of the other parameters?

   Reply: Again we have added tables for all parameter values that should make these easier to find.

4. Panel E/F: The label suggests the plotting of a "cumulative distribution. I am not sure I understand how it is defined. Eq. 4 does not look like a cumulative distribution and the function plotted does not reach one as would be the case for a cumulative distribution function.

   Reply: We agree that the term 'cumulative distribution' was incorrect and have corrected the figure legend.

Figure 4

1. Panel B: This shows that no stable state exists for intermediate P_prod ranges. How was stability defined and determined in this stochastic system? All I could find is a reference to "BifurcationKit package (which tracks steady states using pseudo-arclength continuation)" which is not sufficient. What exactly is the P_prod range for unstable steady states? The lack of axis ticks makes it difficult to read, but eyeballing suggests that maybe around P_prod~125 the system is unstable. However, the simulation depicted in panel K of this figure looks like a stable system.

   Reply: The bifurcation diagram is carried out for the deterministic version of the system since, as discussed above, steady states and their stability cannot be defined for the stochastic system. We note that this is the reason that the region where you see instability in the bifurcation diagram does not correspond to the region where you see limit cycles in the stochastic system. Again, this is due to noise's ability to morph the behaviour of non-linear systems beyond just adding fluctuations around some mean. This is the same phenomenon as we note in Figure 2A, B and is nothing unexpected.

   While we thought it might be helpful to show that a similar transition could be found in a deterministic system, and how this could elegantly be shown in a bifurcation diagram, we recognize that this might instead have been confusing. We have removed figure 4B and instead moved it into a supplementary figure (SFig 17). This also gives us more room to give a thorough explanation of this phenomenon, and the difference between stochastic and deterministic systems.

Figure 5

1. Panel A/B: What are the values of the other parameters? What is the distinctness -- how is it defined and what does it mean? At the very least the name of the variable that is plotted and where it is defined needs to be stated. I could not figure out what is plotted even after reading the methods section.

   Reply: We now provide a table of figure parameters. We have also attempted to rephrase our definitions so that they are better described and have added additional information to the figure legend.

Typos

1. Line 88 Typo "noted"
2. Line 147 typo "we sat"
3. Multiple typos between line 266 and the equation "We will primarily be the" and "simualte", "described"
4. Line 269 "nosie"
5. Line 273 "acording"
6. Line 346 "ou" should be out
7. Line 348 typo "p_start"
8. Line 360 typo "if"
9. Typo page 18, caption of Fig 3 Ptress should be P_stress
10. Typo page 18, caption of Fig 3 "behaviorus" + grammar issue. Sentence needs to be fixed to make it is clear!

    Reply: All these typos are now fixed, sorry for missing these!

11. Fig 3 A/B show three lines but they are not the same but a different subset of the four values for kp*P_stress. It would be easier to compare the two panels if all four values of kp*P_stress were indicated in both panels.

    Reply: We have now added the additional lines, completing the full set of four in each figure.

12. Fig 3 E/F The legend indicates four parameters were changed but only three are visible. Maybe the fourth one is invisible because it is covered by another line?

    Reply: Indeed the third one is barely visible due to being obscured by the fourth one (with the exception of in the corner where it can be seen slightly). We now point this out in the text.

13. Fig 5 caption. Typo "light green". Grammar in "For each parameter combination, the distinctness of the single response pulse (A) and stochastic pulsing (B) behaviours, as the parameter P_prod is varied (Methods)".

    Reply: Light green designates the colour of the dot (light green), unfortunately, we do not understand what typo is referenced as we think the colour is indeed light green. We have fixed the grammar in the sentence.

14. Fig 6 Missing scale for eta_{upstream}. And the notation is confusing as previously defined eta and eta_amp are relabelled as eta_core and eta_upstream.

    Reply: Thanks for noting the missing scale, this has now been added back in. We have renamed the labels for the figure so eta_core and eta-upstream are no longer used.

Reviewer 3

Summary

The paper by Loman and Locke investigates a stochastic model of alternative sigma factor network for their ability to generate different types of pulsing dynamics as experimentally observed. As authors nicely summarise the state of the art, it is currently believed that the system produces alternative pulsing behaviour depending on the type of input it receives. However, the authors turn an existing ODE model of the system into a stochastic model using the chemical Langevin framework and show that the system indeed has the capability of exhibiting both behaviour in certain parameter regime. They further show that the interaction with the noise in the input can shape the tendency of the system to exhibit one type of behaviour vs another. Overall, the results nicely explain some recent unexpected experimental results in the system and suggests a series of new experiments. Also, more broadly, the study provides an example of how stochastic modelling of chemical networks can shed light on the type of behaviour they could exhibit. Overall, this is very interesting paper and fits well in the PLOS Comp Biol. It is nicely and clearly written. I particularly liked the fact that the main results section is short and to the point with details fully explained in the methods. I do not have any major comment. I include a few relatively minor points.

Comments

1. The CLE framework brings in intrinsic noise in a particular way. In general, one could expect the noise in the different components of the system be a function of the parameters of the system, levels of mRNA and burstiness of the different

genes. Could the authors provide a longer discussion of the limitations and the generality of the results obtained by the CLE.

Reply: We thank the reviewer for their positive assessment of the article. In reply to their major comment, we now provide a longer discussion of the limitations and assumptions of the CLE, as discussed in detail in our reply to reviewers 1 and 2 above, in both the introduction and the discussion. We have also re-capitulated our key results using simulations with the Gillespie algorithm, broadening the generality of the results of the paper. We have also extended our discussion of the limitations of the model in the discussion, and discussed how future models should consider bursts of transcription and translation.

Typos

1. I have spotted a few typos in the paper, please read the paper another time carefully. For example on Page 8. "We will primarily be [use?] the CLE to simulate the model"

   Reply: Thank you - we have fixed this typo and have carefully gone through the revised text.

Other

1. A link to a github with code would be good. There is a vague statement in the paper about Code availability.

   Reply: A link to the SLCU gitlab page is available with all code required to regenerate the figures of the paper. This is described in the Code Availability section.
