# Supplementary material for: The σB alternative sigma factor circuit modulates noise to generate different types of pulsing dynamics
Source: PLoS Comput Biol. 2023 Aug 4;19(8):e1011265. doi: 10.1371/journal.pcbi.1011265 (PMC10431680; doi:10.1371/journal.pcbi.1011265)
Supplement: Attachment — Submitted filename: loman_locke_2023_reply_to_reviewer_June_2023.pdf [file pcbi.1011265.s027.pdf]

We thank the reviewers for their careful examination of our work and are happy that Reviewer 1 and 3 are now ready to approve publication. We also appreciate Reviewer 2's continued careful comments and helpful suggestions. We address Reviewer 2's comments below.

Reviewer #1: *The authors have carefully revised the manuscript and answered all my questions. I am happy to recommend publication.*

We thank the reviewer for their helpful suggestions and are happy that they now recommend publication.

Reviewer #2: *The authors made extensive changes that have significantly improved the manuscript. Furthermore, the authors' replies have clarified some misunderstandings that I had about their work. However, even the revised and improved version has some significant issues that should to be further improved.*

We are grateful that the reviewer feels the manuscript is much improved. We address their remaining concerns below.

For a start, the authors should heed the previously expressed advice of referee #3 to "please read the paper another time carefully". This still applies even after this round of revisions. Just to highlight two egregious examples: 1) the supplemental file currently has all references to the bibliography and cross-references replaced by question marks. 2) the rewritten key definitions of the pulse amplitudes now contain mistakes that mix up the transient and asymptotic phases. For example, "The maximum asymptotic pulse amplitude... is measured as the maximum activity of the simulation in the transient phase." The peer review process should not be a substitute for careful proofreading and pre-submission quality control.

We are very sorry that these errors slipped in and agree that it should not be the reviewer's job to spot them. Thank you for taking the time to find them. We have fixed these and other errors in the revised text.

The main focus of my review are the used methods. I have no issue with the CLE now that the authors have shown that Gillespie simulations exhibit the same behaviour. But I think the authors would be well advised to clarify more what their "behavioural measures" are and what they mean. The authors' reply clarified that I incorrectly thought that in Fig. 2 the "Single response pulse magnitude" referred to the same thing as the "amplitude of the single response pulse". I suspect I will not be the only reader confused by the terminology because the terms amplitude and magnitudes are often used interchangeably. (For example, in this manuscript the authors themselves use noise magnitude and noise amplitude

interchangeably.) It is thus problematic to redefine a pulse amplitude into a pulse magnitude by rescaling it by another amplitude. This is not a "mere issue of terminology" but is about an important scientific ingredient that is crucial to understand all the authors' results, namely what exactly the "Single response pulse magnitude" and the ""Stochastic response pulse magnitude" measure. The authors would be well advised to illustrate this key definition in the main text. One way would be, e.g., to promote the supplemental figure SFig. 4 to the main text.

We are happy that the reviewer has no issue with the CLE in the revised manuscript. We also agree that the terminology can be improved. We now consistently use the words *amplitude* and *magnitude* to refer to distinct terms. Furthermore, we have included SFig4 as new panels of Figure 2 (new Fig 2 C and D). We believe that this makes the paper easier to understand and thank the reviewer for the suggestion.

The problem of defining response magnitudes that are not directly amplitudes is compounded by the fact that much of subsequent results are for quantities further derived from these magnitudes, e.g., from Msrp and Msp the authors define Msrp as max(0, Msrp-Msp). The authors should explicitly demonstrate that such derived measures are meaningful. For example, is a system with Msrp*=0 because because Msrp=5 and Msp=7 really a much more stochastic response then say a Msrp=12 and Msp=10 system? This is relevant because the "phase space" analysis rests on quantities further derived from Mrsp* depends on parameters.*

While we agree that the measures can be described clearer (which we now have improved further, see other comments), we believe that this specific example is partially a misunderstanding. For parts of the analysis we only wish to determine how strongly the system exhibits a behaviour, here we use our measures M. The measure M* (or the measures that are derived from it, such as D_srp,sp) is only used in analysis where we wish to determine how *distinctly* the system may generate one behaviour only, e.g. if it produced a stochastic pulsing response *rather than a* single response pulse. For this case our definition of M* makes sense.

To me it seems the key scientific contribution of this manuscript lies in determining a "phase space" of behaviour. In particular, to quantify how the system behaves as a function of the p_prod. But such an analysis strictly relies on the reported quantities to be meaningful. For example, one thing to discuss more explicitly is how these measures characterize/quantify oscillatory systems. When the system oscillates I'd expect for the stochastic pulsing magnitude (SPM) to be around 2 or 3. I suspect the authors don't deem that a relevant

magnitude but they should argue that explicitly. Similarly, the SPM seems to be maximal when there is exactly one stochastic pulse in the time-interval [5,200]. And as soon as a second or third one appears the SPM measure severely drops. It would be good to describe/comment on this.

We thank the reviewer for suggesting adding more discussion of the meaning of the measures. We agree that oscillatory systems can result in a SPM of around 2 or 3, and that an SPM value of 2 is low (as this measure typically reaches at least 50 for simulations in the stochastic pulsing regime). In terms of the SPM being maximal for a single pulse and then dropping severely for more pulses, we note that additional pulses to the first one make little contribution to the mean activity, and so do not cause a severe drop. Moreover, while a single asymptotic pulse could maximise the stochastic pulsing measure for a single simulation (depending on the amplitude of the pulse), the measure is typically taken over a large number of simulations (n > 50). Here, if one simulation has only a single stochastic pulse, others will likely have zero, bringing the mean value of the SPM down. To give the reader additional intuition of these measures, we now discuss these cases in Lines 518-532.

Regarding the single response pulse magnitude I raised a technical question in the last review that I am not sure is entirely addressed by the authors' response. Their pulse distinction is based on an early/transient time interval [0,5] and a late asymptotic one. But that only makes sense if there aren't any stochastic pulses within [0,5], i.e. that the early interval only ever contains either 0 or 1 but never 2 pulses. Could the authors verify that this is indeed the case? The current statement is a bit unclear on this at it seems to talk about the length of the initial pulse, which indeed seem much shorter than that interval. "Finally, to ensure that the transient phase indeed never surpasses 5 hours, we made an automatic scan of a large number (10, 000) of random parameter sets of our model. None of these exhibited an initial pulse longer than 5 hours."

Thank you for the opportunity to clarify our choice of a 5 hour window. We chose a 5 hour window to ensure that the first pulse was captured, not so that other pulses could not be captured within the transient phase. We then measure the likelihood of further pulses using our measurement in the asymptotic phase (5-200 hours). To demonstrate that using 5 hours, and not a shorter duration, for the transient phase, does not affect our conclusions, we performed scans of behaviour space using both a 3-hour and a 5-hour transient phase (Reviewer Figure 1). This demonstrates that the choice of time interval only has minor effects on the measure. We also describe in the methods that setting the transient interval to *(0.0,5.0)* is primarily to ensure that the first pulse is captured captured

(Lines 511-517).

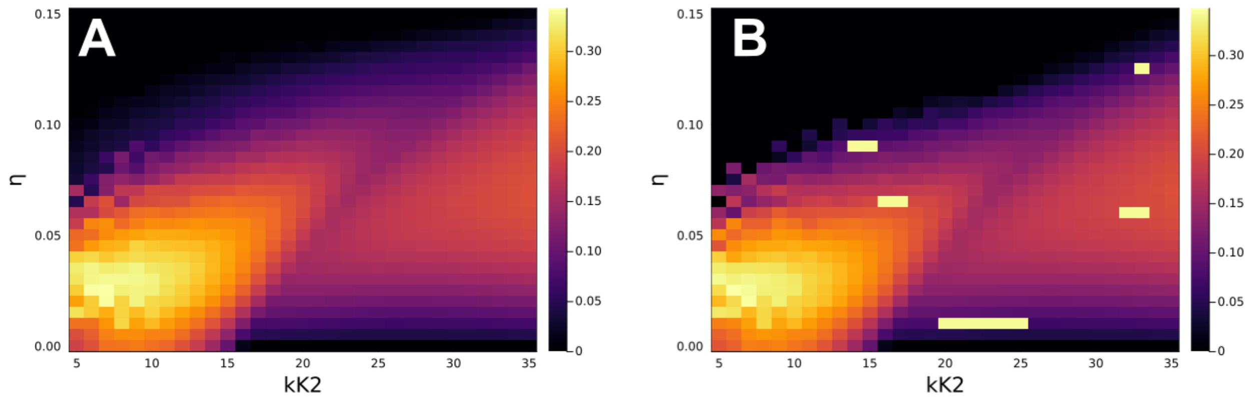

Reviewer Figure 1: In SFig 7 we scan* kK2-eta *space to determine the system's ability to robustly generate both behaviours as* pStress *is varied* (Dsrp,sp)*. This figure is replicated in A. (B) Here we redo the scan from A, but using a 3 hour transient phase instead of a 5 hour one. We note that the scan looks very similar to A, and the slight differences would not affect any of our conclusions. The beige spots are due to the scan not completing for certain parameter values (due to the limited availability of HPC time).

To me it seems that the authors' main finding is that in their modelled system noise can induce stochastic pulsing in a regime before the system becomes oscillatory. Such behaviour is interesting and important. But the authors could embed their findings in the existing literature on such noise induced oscillations and stochastic pulses when a stability analysis of the deterministic system would suggest a single stable fixed point [e.g. their results and how they relate to references 55,56,57 could be discussed in the discussion]. It also seems to be that the stability analysis of the deterministic Narula model is novel and interesting. In the revised manuscript it has been demoted to the supplement. [Possibly in response to one of my comments about the difference between the unstable region of the deterministic system vs the stochastic one. But instead of hiding this difference in the supplement I think it would be much more interesting/appropriate to discuss it explicitly!]

We did indeed move the deterministic bifurcation diagram to the supplemental figure, in part based on the reviewer's comment but other lab members also found the previous figure confusing. With the diagram in its own supplemental figure, we are able to explain in more detail the differences and were not intending to hide them. We have now further clarified the presence of the supplementary figure. We now mention it and its conclusion in the Results (Lines 225-227). We have also added a paragraph on this in the Discussion (Lines 316-321).

I still found the presentation of the model confusing because it is done in two steps

separated over two section 4.1 and 4.2. That leads to seemingly contradictory statements because in both sections the authors refer to 'the model' and 'the parameters' but the meaning changes between the sections. Would it make more sense to just define the final model the authors analyze? (This would also avoid the repetitive ODE table that is mostly overlap between 4.1 and 4.2.) The authors should of course still explicitly discuss the novel parts introduced in their model compared to the published Narula model. But I think doing so by formally defining two slightly different models is counterproductive.

We thank the reviewer for the suggestion, but believe that the presentation of the model in two steps is important, as both stages of model development are analyzed and considered in the paper. In the revised document we have clarified which model we are talking about in each section to avoid confusion.

Some technical points that should be fixed.
The authors state "to prevent negative numbers in our CLE simulations we use a reflective boundary condition". However, when they describe their numerical method it did not sound like they actually implemented reflective boundary condition, but that their code allows for negative numbers and they employed a 'hack' to deal with the subsequent negative reaction rates by taking absolute values of the rates in the stochastic term. "The CLE reflective boundary condition is implemented by, for every term within a square root, replacing it with its absolute value."

Employing an absolute value in the CLE is a well established technique (e.g ref 54). We have now changed our wording, clarifying how the technique is used, rather than referring to it as using a reflective boundary condition.

Below is a (probably incomplete) list of typos that I noticed throughout the manuscript. I implore the authors to take more care in their preparation of future revisions. Peer review should not be used as a substitute for proofreading.

Author summary
Typo: bet-bedge

Main text typos
Line 117 "We note that the CLE and Gillespie algorithm both models intrinsic noise only". models -> model
Line 296 "Ornstein-Uhlenbreck process " Uhlenbreck -> Uhlenbeck
L385 magntiude
L447, 450 Fix quotation marks.

L454 implemenpted
L458 pushes
L477 "The amplitude of the transient pulse. This is measured as the maximum activity of the simulation in the assymptotic phase (t in (0.0, 5.0))."
I suspect the authors mean transient rather than assymptotic [sic] here. Similarly, in the next definition: "The mean asymptotic activity. This is measured as the mean activity of the simulation in the transient phase (t in [5.0, 200.0])." I suspect the authors mean asymptotic rather than transient here.
L524 change -> changes

Fig 4 caption "A similar transition, but generated through Gillespie algorithm simulations, can be found in Supplementary figure 19"
I suspect the authors mean Supplementary Fig 18.
Fig 4 caption "12 different selected values of pprod (used in C-N)"
I think the authors mean B-M in the current iteration of this figure.

ODE systems: in first line v0 should be v_0

In general, the authors correctly follow the convention of separating numbers and units with a space and non-italicized units. But sometimes they don't. The presentation should be made consistent.

Supplement
"subsitutions "
SFig 4 caption typo "asymtotic" should be asymptotic, "the the" should be the.
SFIg 5. "upstreams" should be upstream
SFIg 2 axis label typo "stady" should be steady
SFig 7 caption "this examples"
All references and cross-references currently are ? or ??.

We thank the reviewer for spotting these typos, they are now fixed.

Reviewer #3: The authors have fully revised the paper addressing my concerns.

We thank the reviewer for their helpful suggestions and are glad that they are now happy to recommend publication.
